# High natural PHA production from acetate in *Cobetia* sp. MC34 and *Cobetia marina* DSM 4741^T^ and in silico analyses of the genus specific PhaC_2_ polymerase variant

**DOI:** 10.1186/s12934-021-01713-0

**Published:** 2021-12-20

**Authors:** Mikkel Christensen, Piotr Jablonski, Bjørn Altermark, Knut Irgum, Hilde Hansen

**Affiliations:** 1 Department of Chemistry, UiT-The Arctic University of Norway, 9037 Tromsø, Norway; 2 Department of Chemistry, Umeå University, 90187 Umeå, Sweden

**Keywords:** *Cobetia* spp., *Halomonas* spp., *Halomonadacea*, Polyhydroxyalkanoates, PHA, PHB, PHBV, PhaC, Acetate, Genome

## Abstract

**Background:**

Several members of the bacterial *Halomonadacea* family are natural producers of polyhydroxyalkanoates (PHA), which are promising materials for use as biodegradable bioplastics. Type-strain species of *Cobetia* are designated PHA positive, and recent studies have demonstrated relatively high PHA production for a few strains within this genus. Industrially relevant PHA producers may therefore be present among uncharacterized or less explored members. In this study, we characterized PHA production in two marine *Cobetia* strains. We further analyzed their genomes to elucidate *pha* genes and metabolic pathways which may facilitate future optimization of PHA production in these strains.

**Results:**

*Cobetia* sp. MC34 and *Cobetia marina* DSM 4741^T^ were mesophilic, halotolerant, and produced PHA from four pure substrates. Sodium acetate with- and without co-supplementation of sodium valerate resulted in high PHA production titers, with production of up to 2.5 g poly(3-hydroxybutyrate) (PHB)/L and 2.1 g poly(3-hydroxybutyrate-co-3-hydroxyvalerate) (PHBV)/L in *Cobetia* sp. MC34, while *C. marina* DSM 4741^T^ produced 2.4 g PHB/L and 3.7 g PHBV/L. *Cobetia marina* DSM 4741^T^ also showed production of 2.5 g PHB/L from glycerol. The genome of *Cobetia* sp. MC34 was sequenced and phylogenetic analyses revealed closest relationship to *Cobetia amphilecti*. PHA biosynthesis genes were located at separate loci similar to the arrangement in other *Halomonadacea*. Further genome analyses revealed some differences in acetate- and propanoate metabolism genes between the two strains. Interestingly, only a single PHA polymerase gene (*phaC*_2_) was found in *Cobetia* sp. MC34, in contrast to two copies (*phaC*_1_ and *phaC*_2_) in *C. marina* DSM 4741^T^. In silico analyses based on *phaC* genes show that the PhaC_2_ variant is conserved in *Cobetia* and contains an extended C-terminus with a high isoelectric point and putative DNA-binding domains.

**Conclusions:**

*Cobetia* sp. MC34 and *C. marina* DSM 4741^T^ are natural producers of PHB and PHBV from industrially relevant pure substrates including acetate. However, further scale up, optimization of growth conditions, or use of metabolic engineering is required to obtain industrially relevant PHA production titers. The putative role of the *Cobetia* PhaC_2_ variant in DNA-binding and the potential implications remains to be addressed by in vitro*-* or in vivo methods.

**Supplementary Information:**

The online version contains supplementary material available at 10.1186/s12934-021-01713-0.

## Background

The Arctic marine environment constitutes a promising source of cold-adapted microorganisms of potential use for biotechnological production of proteins, green chemicals, polymers, or other valuable compounds [1]. Polyhydroxyalkanoates (PHA) are polyester bioplastics that are gaining renewed commercial interest as green replacements of oil-plastics derived from fossil resources, due to attractive material properties resembling common types of oil-plastics such as polyethylene, but with the advantage of biodegradability in the marine environment as determined by the ASTM standard methods [2, 3]. This biodegradability is furthermore an advantage compared to the bioplastic polylactic acid (PLA) that currently has a high share of the global bioplastic market, but requires high temperature to biodegrade in soil and compost [4]. PHA is produced commercially at low volumes (< 30,000 tons per year) compared to PLA (> 300,000 tons per year) and oil-plastics (> 370 million tons per year) [5]. Industrial production of PHA by bacterial fermentation typically utilizes plant-oils or waste lipids from the agricultural industry or various forms of pure and mixed carbohydrates [6]. A PHA polymer is composed of 3-hydroxyalkanoic acid monomers of varying length, where the excess alkyl chains beyond the 3-hydroxy group take the roles of side chains in the linear polyester. The monomers are classified as either short chain length PHA (scl-PHA) of carbon chain lengths C_3_ to C_5_ or as medium chain length PHA (mcl-PHA) of chain lengths C_6_ to C_14_, with increased flexibility for the longer chain length polymers [7]. The chain length- and composition determine, together with the molecular weight, the material properties after extraction and processing into a PHA bioplastic product [8, 9]. Establishing substrate utilization and PHA composition phenotypes thus constitute important objectives when characterizing bacterial strains for PHA production.

PHA was discovered as the constituent of intracellular granules found in a Gram-positive *Bacillus* species, but are now known to be prevalent also in *Archaea, Cyanobacteria* and in Gram-negative α-, ß-, γ- and δ-*proteobacteria* [10]. The biological function is generally described as a response to nutrient availability such as when excess carbon is available relative to nitrogen, which was demonstrated already in the 1950s [11]. Recent studies have found several other fitness enhancing effects due to accumulation of PHA in bacteria. These include protection against reactive oxygen species [12] and DNA damage from exposure to UV light [13, 14], and protection against osmotic shock, temperature change, and heavy metals [15]. Ecological studies of the Arctic environment found PHA producing bacteria to be present based on staining with lipophilic dyes and amplification of the PHA polymerase gene *phaC*, which supports the existence of selective pressure for PHA accumulation in this extreme environment [16, 17]. Taken together, these findings point at cold- and saline environments such as the Arctic marine environment as promising reservoirs for finding novel PHA producing strains of potential use in biotechnology.

Biosynthesis of PHA requires three enzymes utilizing precursors originating from the tricarboxylic acid (TCA) cycle, beta-oxidation-, fatty acid degradation-, and other organism specific pathways [18]. Synthesis of the most common type of PHA, the C_4_ poly(3-hydroxybutyrate) (PHB), starts with condensation of two acetyl-CoA molecules by the acetyl-CoA acetyltransferase (ß-ketothiolase) PhaA into acetoacetyl-CoA. Subsequent reduction by acetoacetyl-CoA reductase PhaB produces (R)-3-hydroxybutyryl-CoA, which is polymerized into PHB by polyhydroxyalkanoate polymerase PhaC, encoded by the *phaC* gene. Four different classes of PhaC polymerases are generally described and classified based on their subunit composition and the ability to incorporate scl- or mcl-hydroxyalkanoate precursors, with a fifth class recently proposed primarily based on phylogenetic analyses [19, 20]. A type of proteins known as phasins (PhaP) control size and coagulation of the hydrophobic PHA granules [21], and coats the outer layer together with PhaC, the PHA synthesis repressor PhaR and other PHA regulatory proteins such as the PHA depolymerase enzyme PhaZ. This complex of granules and proteins together form an in vivo subcellular organelle referred to as the carbonosome [22].

The Gram negative γ*-proteobacterial* family *Halomonadaceae* consists of 13 genera which are characterized as halotolerant or halophilic, and are typically isolated from extreme environments such as salt lakes [23, 24] or from the marine environment, including the Arctic [25]. Several species of the genus *Halomonas* are well established as producers of scl-PHA and envisioned as promising candidates for biotech production due to their salt- and alkali tolerance and the applicability of genetic engineering techniques used for strain optimization [26].

Closely related to the *Halomonas* genus is the *Cobetia* genus, with five species currently being recognized: *Cobetia marina* [27], *Cobetia pacifica*, *Cobetia amphilecti and Cobetia crustatorum* [28], and *Cobetia litoralis* [29]. The type strain *C. marina* DSM 4741^T^ has changed phylogenetic classification several times since its original discovery and classification as *Arthrobacter marinus* in 1970 [30] and later as *Pseudomonas marina* [31], *Deleya marina* [32] and *Halomonas marina* [33]. Earlier phylogenetic investigations of *Cobetia* and *Halomonas* show polyphyletic lineages based on 16S rRNA sequences [34]. Current species classifications of *Cobetia* are based on DNA hybridization and phenotypic assays [28], however, several strains without species designation are deposited in the National Center for Biotechnology Information (NCBI) database. Strain specific metabolic differences such as temperature tolerance, substrate utilization, and the requirement of sodium ions for growth have been described for strains of *C. marina* found in three different marine habitats [35]. Despite PHB positive type strain designations for *C. marina* [27], *C. crustatorum* [29], *C. amphilecti*, *C. litoralis*, and some strains of *C. pacifica* [28], only a few *Cobetia* strains have been thoroughly investigated for PHA production. The strain *Halomonas* sp. SF2003, previously named *Cobetia* sp. i.4786 [36, 37], is demonstrated to produce PHB from agro-industrial waters rich in sugars and the co-polymer poly(3-hydroxybutyrate-*co*-3-hydroxyvalerate) (PHBV) when supplied with valeric acid as co-substrate [36, 38]. Similarly, *Halomonas marina* HMA 103 isolated from a solar saltern in India is capable of producing PHB and PHBV [39]. Most recently, several newly isolated *Cobetia* strains without formal species designations are demonstrated to produce PHA from polysaccharide substrates originating from brown- and green algae biomass [40, 41]. This demonstrates the potential of using different strains of *Cobetia* for PHA production.

The aim of this study was to analyze and characterize PHA production for two marine isolates, *Cobetia* sp. MC34 and the genus type-strain *C. marina* DSM 4741^T^. Both strains were found to be putative PHA producers by fluorescent microscopy and Fourier transform infrared spectroscopy (FT-IR) analyses of cells grown on agar plates supplemented with carbon sources. The impact of carbon source, temperature and salinity on growth rate and putative PHA production was analyzed after cultivation in liquid cultures. PHA production was verified and quantified using Gas Chromatography-Flame Ionization Detector (GC-FID) analysis, and the PHA polymer structures were confirmed by Nuclear Magnetic Resonance (NMR) spectroscopy. The genome of *Cobetia* sp. MC34 was sequenced to enable allocation of *Cobetia* sp. MC34 to species level, and to allow identification of PHA biosynthesis- and acetate metabolism genes which may form the basis for future targeted studies of PHA metabolism in these strains. Lastly, we performed an in silico study of the *Cobetia* genus specific PhaC_2_ enzyme with an intriguing C-terminal end to shed light on its putative function.

## Methods

### Bacterial strains and media recipes

*Cobetia* sp. MC34 was isolated in May 2017 from an environmental swab taken from a maturation room for salted fish at a fish-landing facility outside of Tromsø, Norway. The genus type strain *C. marina* DSM 4741^T^ originally isolated from Woods Hole, USA [30], was obtained from DSMZ (Cat. no. DSM4741). All culture stocks were stored at − 80 °C in 20% glycerol in M4 media and revived on M4 or Marine agar (Difco 2216). The M4 medium contained (per liter 0.2 µm filtered seawater mixed 1:1 with Milli-Q water): 1 g glucose, 1 g malt extract (Merck), 1 g yeast extract (Merck), 1 g peptone (Fluka), and 1 mL glycerol (VWR). The pH of the M4 medium was adjusted to 8.2 using potassium hydroxide before autoclaving. Solid medium was prepared by adding 15 g/L of agar (VWR). MMY agar plates with 1 g/L yeast extract (Merck) was made according to a previously described synthetic seawater medium [42]. Since growth of *Cobetia* sp. MC34 appeared hampered by development of acidic conditions, two bicarbonate pH buffered PHA production media (MMCY and MMCY_2) were developed. The autoclaved MMCY media contained (per litre): 1 g yeast extract (Merck), 12.9 g sodium chloride, 4 g sodium sulfate, 0.2 g potassium dihydrogen phosphate, 1.4 g magnesium chloride, 0.5 g potassium chloride, 0.25 g ammonium chloride, and 0.112 g calcium chloride. The room tempered media was supplemented to a final concentration of 4 g/L of autoclaved sodium bicarbonate, and sterile filtered vitamin- and trace element solutions as described elsewhere [42]. The MMCY_2 media was made similarly, except that sodium chloride was eliminated and only 2 g/L of sodium bicarbonate was added, to compensate for the addition of sodium from the use of sodium acetate with this media.

### Agar plate based screening by fluorescent microscopy and Fourier transform infrared spectroscopy (FT-IR)

A single colony of revived bacteria was re-streaked on MMY agar plates and MMY agar plates supplemented with either 2% (20 g/L) glucose or 2% glycerol (vol/vol). Cells were scraped off after 72 h incubation at 14 °C and re-suspended in 2.5% glutaraldehyde in 1 × PHEM buffer (60 mM PIPES (VWR), 25 mM HEPES (VWR), 10 mM EGTA (Sigma-Aldrich), 4 mM MgSO_4_·7H_2_O, pH 6.9), stained with 5 µg/mL Nile-Red (Sigma-Aldrich) for 30 min at room temperature followed by centrifugation at 11,000×*g* for 1 min. The supernatant was removed and the cells washed twice and re-suspended in 2.5% glutaraldehyde in 1 × PHEM buffer, mounted on agar pads [43] and viewed in a ZEISS Axio Observer 7 microscope with a 100 × NA 1.46 alpha PlanApo oil immersion objective. Images were taken by an Axiocam 506 camera using Colibri LED-fluorescence at wavelengths 559 nm (excitation) and 636 nm (emission). The fluorescent intensity of the images was normalized to 1500 using the ZEN 3.2 software (Carl Zeiss Microscopy GmbH). Cells from the same plates were analyzed by FT-IR as described in the “FT-IR analysis” section.

### Cultivation conditions for small-scale liquid cultures

The influence of temperature on the specific growth rate and putative PHA production were tested in 5 mL cultures using MMCY media supplemented with 2% (20 g/L) glucose and incubated at 4 °C, 14 °C, 20 °C, 25 °C, 30 °C, 37 °C, and 42 °C, at 200 rpm. Pre-cultures were prepared in triplicate from single colonies of *Cobetia* sp. MC34 or *C. marina* DSM 4741^T^ into 3 mL M4 media with overnight incubation at room temperature (≈ 25 °C, 1270 shakes per minute on a Heidolph Multi Reax shaker), before centrifugation at 11,000×*g* for 1 min in Eppendorf tubes and re-suspension to a start OD of 0.05 in MMCY media. The specific growth rate, *k*, was calculated from linear plots corresponding to the equation

1 $$k = {{Ln\left( {\frac{{OD_{600\left( 2 \right)} }}{{OD_{600\left( 1 \right)} }}} \right)} \mathord{\left/ {\vphantom {{Ln\left( {\frac{{OD_{600\left( 2 \right)} }}{{OD_{600\left( 1 \right)} }}} \right)} {\left( {T_{2} - T_{1} } \right)}}} \right. \kern-\nulldelimiterspace} {\left( {T_{2} - T_{1} } \right)}}$$

where OD_600(x)_ is the optical density at 600 nm corresponding to the time point T_x_, sampled during the exponential phase at 2 h intervals. The putative PHA production was measured by FT-IR analysis (as described in “FT-IR analysis” section) when the cultures reached stationary phase, and normalized to production per hour. The results are presented as the average values between duplicate experiments, with error bars depicting the coefficient of variation.

### Cultivation conditions for shake-flask production of PHA

Pre-cultures were grown similarly as described for small-scale liquid cultures, except that overnight 3 mL cultures were re-suspended in 50 mL M4 media and grown at 30 °C, 200 rpm, until reaching mid- to late exponential phase (OD_600_ of 3–4), followed by centrifugation at 11,000×*g*, 20 °C for 10 min. The pellets were re-suspended in MMCY and MMCY_2 media to a start OD_600_ of ≈ 0.15 in triplicate 80 mL cultures grown in 250 mL baffled shake-flasks incubated at 30 °C, 200 rpm. Sodium acetate was supplemented to MMCY_2 media (2%, 20 g/L) while glycerol (1.5% vol/vol), glucose and fructose (4%, 40 g/L) were supplemented to MMCY media. Sodium valerate, prepared by adding an equimolar amount of sodium hydroxide to valeric acid, C_5_, (99%, Alfa Aesar), sodium hexanoate, C_6_, and sodium octanoate, C_8_, (TCI) were added to the media at 8 mM concentrations where indicated. At the end of the experiment, 40 mL of bacterial culture was harvested by centrifugation at 13,000×*g*, 4 °C for 15 min and washed twice in 25 g/L sodium chloride before freezing and lyophilization for determination of cell dry weight (CDW) and subsequent extraction and quantification of PHA (as described in “Quantification of PHA by Gas Chromatography-Flame Ionization Detector (GC-FID)”).

### FT-IR analysis

Semi-quantitative analysis of PHA by FT-IR has been demonstrated for mixed cultures of bacteria using a simple model that employs the ratio between the peak areas of the carbonyl-ester and the amide band I when the PHA content is below 44% of the CDW [44]. Bacterial cells for FT-IR analysis were either scraped off from agar plates using an inoculation loop, re-suspended in 96% ethanol and spun down in Eppendorf tubes at 21,000×*g* for 1 min, or 500 µL were taken from liquid cultures and spun down similarly followed by re-suspension of the pellet in 96% ethanol and a second spin to subsequently remove the supernatant. Spectral information was collected for the full range (650–4000 cm^−1^) with 64 background scans and 256 sample scans using HappGenzel apodization without zero fill factor on an Agilent Cary 630 ATR FT-IR instrument controlled by Agilent MicroLab PC software. The putative PHA production based on the carbonyl-ester and amide band I-ester regions was calculated as the ratio between the areas of their corresponding spectral regions (C:A 1 ratio) defined as 1763–1705 cm^−1^ (carbonyl-ester) and 1705–1580 cm^−1^ (amide band I-ester). The C:A 1 ratio was calculated from raw spectra using the integral function, while spectra obtained from agar plates were baseline corrected to remove negative absorbance values using SpectraGryph, Software for optical spectroscopy v. 1.2.15 (Dr. Friedrich Menges, Oberstdorf, Germany).

### Quantification of PHA by Gas Chromatography-Flame Ionization Detector (GC-FID)

The amount of PHA in bacterial pellets was quantified by GC-FID after methanolysis in a 1:1 (vol/vol) mixture of chloroform and 15% concentrated sulfuric acid dissolved in methanol. Approximately 15–30 mg of lyophilized cells were added to 3 mL of the methanolysis mixture containing 0.2% benzoic acid as internal standard, and reacted for 12 h at 100 °C. PHBV (Sigma-Aldrich) was used as external standard. After the reaction, 1.5 mL of Milli-Q water was added to separate the phases followed by careful transfer of the organic phase into GC analysis vials. The samples were analyzed by an Agilent 7820A GC-FID system (Agilent Technologies) using a 30 m long by 0.32 mm i.d. DB-5MS column with 0.25 μm film thickness. The injector temperature was set to 250 °C with a split ratio of 10:1 and the injection volume was 1 μL. The temperature program was set to 50 °C with hold for 5 min, then a linear increase to 250 °C at a rate of 15 °C/min, followed by increase to 310 °C at a rate of 30 °C/min. Hydrogen was used as carrier gas at 0.8 mL/min flow with a column head overpressure of 37 kPa. At least two technical replicates for each PHA extraction were injected.

### Nuclear magnetic resonance (NMR)

PHA from approximately 50 mg of lyophilized cells was extracted in screw-cap culture tubes with 2 mL of chloroform at 100 °C for 1 h. The extracts were then allowed to cool to room temperature, filtered through 0.22 μm nylon syringe filters, and dried under a stream of nitrogen gas at room temperature. The dried samples were then dissolved in 0.5 mL CDCl_3_ (99.8% atom-D, Acros). ^1^H NMR and ^13^C NMR spectra were recorded at 298 K with a Bruker DRX-400 spectrometer at 400 MHz and 100 MHz, respectively.

### Statistical analysis

The coefficient of variation (standard deviation) for the specific growth rate and the C:A 1 ratio from 5 mL cultures was calculated in Microsoft Excel 2016 using the STDEV.P function from average values of two experiments with 3 biological replicates each. The coefficient of variation (standard deviation) of OD_600_, the CDW and the PHA content was calculated from a total of 6 biological replicates obtained from two independent experiments using Pandas v. 1.1.3 in Python v. 3.8.8.

### DNA extraction and 16S rRNA PCR

DNA from *Cobetia* sp. MC34 was extracted from an overnight culture using DNeasy Blood & Tissue kit (Qiagen) following the manufacturers’ instructions. The partial 16S rRNA sequence for *Cobetia* sp. MC34 was amplified by PCR using 10 mM each of the universal bacterial primers fD1/27F (5′AGAGTTTGATCCTGGCTCAG) and rP2/1492R (3′TACGGYTACCTTGTTACGACTT) [45], 10 mM dNTP and 1.25 U Taq polymerase in 1 × Standard Taq buffer (New England Biolabs). Five nanograms of DNA was added as template and the reaction was cycled 30 times (94 °C for 30 s, 55 °C for 30 s and 72 °C for 2 min). The PCR product was treated with 2 µL ExoSap-IT (Affymetrix®, Thermo Fisher) for 15 min at 37 °C followed by 15 min at 80 °C. The PCR product was then sequenced in one direction using primer 515F (5′-GTGCCAGCAGCCGCGGTAA-’3) and BigDye™ Terminator v3.1 Cycle Sequencing Kit following the manufacturers’ instructions (Applied Biosystems™). The full length 16S sequence was later obtained from the annotated whole genome sequence.

### Genome sequencing, assembly and annotation

Extracted DNA was eluted in 10 mM Tris–HCl pH 8 and the quality and quantity measured on a Qubit fluorimeter (Invitrogen). Sequencing of the genome was done at The Norwegian Sequencing Centre, Oslo, Norway, using Nextera™ DNA Flex Tagmentation and a Illumina MiSeq. The paired end sequencing reads were quality checked and analyzed for presence of adapter sequences using FastQC [46] and subsequently trimmed with first Trimmomatic v.0.39 (Settings: Phred33, Headcrop 15, average quality threshold 29, minlen 100) [47] and secondly to remove the Nextera specific adapter sequences using BBduk v. 38.22 (Settings: ftm 5, Ktrim R, K 23, Mink 9, Hdist 1, Tbo and Tpe Yes). Kraken2 v. 2.1.1 was used for contamination analysis [48, 49]. The sequencing reads were normalized and error-corrected with BBnorm (Settings: Prefilter true, target 50, min 5 and error correction settings ecc t, keepall passes 1, bits 16 prefilter) and merged with BBmerge [50]. Merged and unmerged reads were assembled using SPAdes v. 3.13.0 run in Python v. 2.7.15 [51]. The assembled contig files were evaluated for genome completeness using BUSCO v. 4.0.4 database oceanospirillales_odb10 [52] and evaluation of genome assembly parameters (number of contigs, N50, etc.) were done with Bandage and Quast v. 5.0.2 [53, 54]. Sequence reads were mapped back onto the assembled genome using Bowtie2 v. 7. 3. 0 [55] and alignment statistic was generated with Samtools v. 1.9 [56]. The assembled genome was first annotated with Prokka v. 1.13 [57] and later, for publishing, with the NCBI Prokaryotic Genome Annotation Pipeline [58]. This Whole Genome Shotgun project has been deposited at DDBJ/ENA/GenBank under the accession JADPPY000000000. The version described in this paper is version JADPPY010000000. The sequencing reads are deposited under the NCBI BioProject accession PRJNA678481 and in the NCBI Sequence Read Archive under accession SRR13530190.

### Phylogenetic analyses by 16S rRNA and Average Nucleotide Identity (ANI) matrix

The 16S rRNA sequences (Additional file 1: Table S1) were obtained from either NCBI’s 16S RefSeq database (https://www.ncbi.nlm.nih.gov/refseq/targetedloci/16S_process/), the SILVA ribosomal RNA database project (https://www.arb-silva.de/) or from whole genome sequences obtained from Genbank’s Genome Database (https://www.ncbi.nlm.nih.gov/genome/). The *C. necator* 16S sequence was obtained from NCBI (NR_102851).

The Muscle [59] algorithm (Settings: Gap open -1000, gap extend -1.2, neighbour-joining (NJ) clustering) in MEGA7.0.26 was used to align the 16S rRNA sequences and the Maximum Likelihood (ML) method were used to construct the 16S tree (Settings: Gamma distributed with 2 discrete categories (+ G, parameter = 0.0500), partial deletion with 75% cut-off value and nearest neighbour interchange with default setting for initial tree search) [60, 61]. Evolutionary distances were determined using the Kimura 2-parameter method [62] and the phylogeny tested with 1000 bootstrap replicates [63]. The final tree contains 1520 positions and was visualized using Itol v6 [64].

The ANIb algorithm in PyAni software v. 0.2.10 [65] in Python v. 3. 9. 1 was used to align the whole genome sequences (Additional file 1: Table S1) and generate an ANI matrix used to construct a dendrogram using Pythons Plotly package v. 4. 12. 0 in Python v. 3. 8. 8.

### Genome searches

The Basic Local Alignment Search Tool (BLAST) [66] function in CLC MainWorkBench v. 20.0.3 (Settings: Blosum62 matrix, gap cost 10 and 1, word size 2, expect 1.0 and mask low complexity regions) was used to query both DNA- and protein sequences against translated *Cobetia* genome sequences (Additional file 1: Table S1). PhaZ from *Pseudomonas oleovorans* (P26495) were obtained from The UniProt Consortium 2020 (https://www.uniprot.org/) while PhaZ from *H. bluephagenesis* TD01 (EGP18355.1, EGP19590.1, EGP20509.1), and PhaC from *Halomonas* sp. R5-57 (CEP36398.1 and CEP35484.1), *H. bluephagenesis* TD01 (EGP20415.1 and EGP19504.1) and *Halomonas* sp. O-1 (BAO57489.1 and BAO57490.1) were obtained from Genbank (https://www.ncbi.nlm.nih.gov/genbank/). *Cobetia* spp. *phaC* sequences were obtained from either the annotated whole genome sequences or from BLAST [66] queries against the translated genomes (Additional file 1: Table S1) using *C. marina* DSM 4741^T^ PhaC_1_ and PhaC_2_. The Kyoto Encyclopedia of Genes and Genomes (KEGG) BlastKOALA v. 2.2 (https://www.kegg.jp/blastkoala/) was used for metabolic pathway analysis based on whole genome sequencing data [67].

### PhaC_2_ in silico analysis

The *Cobetia* sp. MC34 PhaC_2_ amino acid sequence (WP_213113863) was used as template to the PSIPRED server v. 4.0 (http://bioinf.cs.ucl.ac.uk/psipred/) which generated the secondary structure prediction and amino acid composition graphic [68, 69]. The *Cobetia* sp. MC34 PhaC_2_ was searched against NCBI’s non-redundant protein sequences using protein–protein BLAST algorithm (Protein BLAST) (https://blast.ncbi.nlm.nih.gov/Blast.cgi?PROGRAM=blastp&PAGE_TYPE=BlastSearch&LINK_LOC=blasthome) [66]. *Cobetia* and *Halomonas* PhaC_2_ sequences were searched for domain hits against NCBI’s Conserved Domain Database v. 3.19 (NCBI CDD) using CD-Search (https://www.ncbi.nlm.nih.gov/Structure/cdd/wrpsb.cgi) and Batch CD-search (https://www.ncbi.nlm.nih.gov/Structure/bwrpsb/bwrpsb.cgi) with the settings “low-complexity filter” and “Composition based statistics adjustment” off [70–74]. Molecular weight (MW) and isoelectric point (pI) values were calculated using Expasy Compute pI/MW tool (https://web.expasy.org/compute_pi/, SIB Swiss Institute of Bioinformatics).

## Results

### Genome sequencing of *Cobetia* sp. MC34

*Cobetia* sp. MC34 was genome sequenced in order to identify and compare PHA biosynthesis genes with the genome sequenced *C. marina* DSM 4741^T^ [75], and to resolve the evolutionary relationships within the *Cobetia* genus. The Illumina Paired-end sequencing of *Cobetia* sp. MC34 generated 1,437,981 raw reads of 301 bases in both directions, which was reduced to 1,010,167 reads after trimming, normalization and merging, plus 38,293 unmerged reads from both directions. The assembled draft genome contained a total of 4,003,645 base pairs (bp) with an N50 of 111,054 bp and a GC content of 62.58%. The genome assembly consisted of 175 contigs exceeding a length of 200 bp, and 109 contigs exceeding a length of 500 bp. The genome contained 3487 genes, of which 3399 were predicted to be protein coding. The quality of the genome assembly was evaluated for contamination and completeness by Kraken2 analysis, which showed contamination to be not present. Busco’s *Oceanospirillales* database contains 619 essential genes, of which 618 complete single copy orthologs and only one fragmented were found in *Cobetia* sp. MC34. Hence, the draft genome of *Cobetia* sp. MC34 was of sufficient quality for identification of PHA biosynthesis genes and phylogenetic studies.

### *Cobetia* sp. MC34 is a strain of *Cobetia amphilecti,* with close relationship to the PHA producer *Halomonas* sp. SF2003

BLAST analysis of the complete 16S rRNA gene of *Cobetia* sp. MC34 revealed high similarity (> 99% identity) to several species annotated as *Halomonas* and *Cobetia* and a Maximum Likelihood (ML) tree was constructed to illustrate the phylogenetic relationship of *Cobetia* sp. MC34 to members of the *Halomonadacea* family. The 29 sequences (Additional file 1: Table S1) included in the ML tree represent the five recognized species of *Cobetia* represented by a total of sixteen *Cobetia* isolates currently available as full genomes, as well as *Halomonas* sp. SF2003 and nine other verified PHA producing strains of *Halomonas*. *Chromohalobacter salexigens* and *Zymobacter palmae* were included to provide reference to a comprehensive *Halomonadacea* phylogeny previously published [34]*.*

**Fig. 1:**
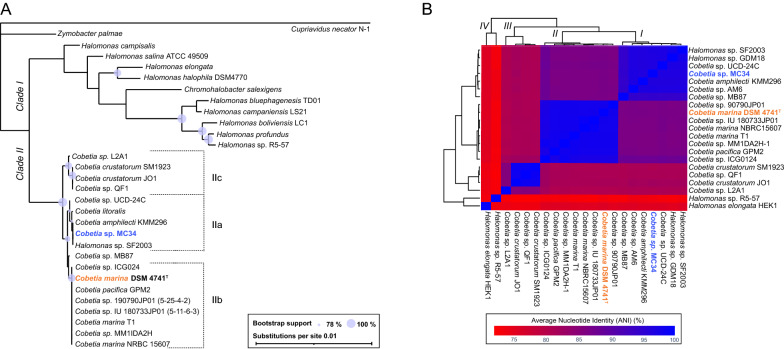
Phylogenetic analyses of *Cobetia sp*. MC34 based on the 16S rRNA gene and the draft genome sequence. **A** The ML-tree based on 16S rRNA gene sequences include recognized species of *Cobetia*, genome sequenced strains of *Cobetia*, known PHA producing *Halomonas* species and two members of the *Halomondacea* family. The 16S rRNA gene from *C. necator* was included as outgroup. Bootstrap support values above 75% (1000 replicates) are depicted as bubbles. The scale bar shows branch length measured as substitutions per site. **B** The Average Nucleotide Identity (ANI) tree was constructed from whole genome sequences. The ANI similarity values are shown as colors from 75% (red) to 100% (blue). *Halomonas elongata* HEK1 and the marine isolate *Halomonas* sp. R5-57 were included as outgroups in the ANI analysis.

As shown in Fig. 1A, two main clades and two outgroups were resolved in the 16S ML tree. Clade I contains *Halomonas sensu stricto* sequences and *C. salexigens* in accordance with earlier studies [34, 76]. Clade II contains three subclades (IIa, IIb and IIc) which include all *Cobetia* species and *Halomonas* sp. SF2003. Despite its name, *Halomonas* sp. SF2003 is shown to belong to the *Cobetia* genus [76]. *Cobetia* sp. MC34 allocates to subclade IIa together with *Halomonas* sp. SF2003 and *C. amphilecti* KMM296. These three isolates as well as *Cobetia* sp. AM6 and *Halomonas* sp. GDM18 (not shown) share identical 16S sequences. *Cobetia litoralis* and *Cobetia* sp. UCD-24C contains only two and three nucleotide substitutions compared to *Cobetia* sp. MC34, respectively, and therefore fell into subclade IIa (bootstrap value of 89%). Subclade IIb (bootstrap value of 96%) is the largest and contains the type strain *C. marina* DSM 4741^T^, strains annotated as *C. marina*, *C. pacifica* GPM2 and five strains without species designation. *Cobetia* sp. MB87 has four nucleotide substitutions compared to *C. marina* DSM 4741^T^, and does not cluster well with the *C. marina* group (bootstrap value of 54%). The two strains of *C. crustatorum* clustered together with *Cobetia* sp. L2A1 and *Cobetia* sp. QF1 in subclade IIc (bootstrap value of 94%).

Whole genome sequence alignment and calculation of the Average Nucleotide Identity (ANI) can increase phylogenetic resolution compared to use of the 16S rRNA gene tree alone [77]. The draft genome of *Cobetia* sp. MC34 was therefore compared with sixteen *Cobetia* genome sequences available in NCBI’s whole genome dendrogram (excluding metagenomes) and the *Halomonas* isolates SF2003 and GPM18 (Additional file 1: Table S1). *Halomonas elongata* HEK1 and the Arctic marine isolate *Halomonas* sp. R5-57 were included as outgroups in the analysis. The genome sequence for *C. litoralis* is currently not available, and the *Cobetia* phylogeny is thus incomplete. Figure 1B shows the resultant ANI tree with four clades that further elucidated the phylogenetic relationship between species of *Cobetia*. Clade I contains *C. amphilecti* KMM296 and *Cobetia* sp. MC34*,* -AM6, *Halomonas* sp. SF2003 and *-*GPM18, *Cobetia* sp. UCD-24C and *Cobetia* sp. MB87. This is the same clustering as observed in the 16S rRNA gene tree (Fig. 1A). All members of this clade except *Cobetia* sp. MB87 showed ANI values between 96 and 98%, suggesting they are strains of *C. amphilecti*, following an ANI species cut-off value of 96% [78]. Closest to the *C. amphilecti* clade is the *C. marina* DSM 4741^T^ clade, also in agreement with the 16S rRNA gene tree. The species split of *C. amphilecti* and *C. marina* is supported by ANI values below 87%. *C. pacifica* GPM2 clusters in the *C. marina* DSM 4741^T^ clade where all members have ANI values above 98%, indicating that they belong to the same species. The third clade contains two strains of *C. crustatorum*, *Cobetia* sp. QF1 and *Cobetia* sp. L2A1, with the latter being most divergent with an ANI value of only 86%. *Cobetia* sp. QF1 and the two *C. crustatorum* strains have ANI values of 99%, indicating *Cobetia* sp. QF1 to be a strain of *C. crustatorum*. The fourth clade contained the two *Halomonas sensu stricto* sequences that separate as outgroups relative to the three other clades.

Thus, the 16S rRNA and ANI analysis suggest placement of *Cobetia* sp. MC34, *Cobetia* sp. AM6, *Halomonas* sp. SF2003, and *Halomonas* sp. GDM18 as strains of the recognized specie *C. amphilecti*.

### Agar plate pre-screening indicate PHA production for *Cobetia* sp. MC34 and *C. marina* DSM 4741^T^

**Fig. 2:**
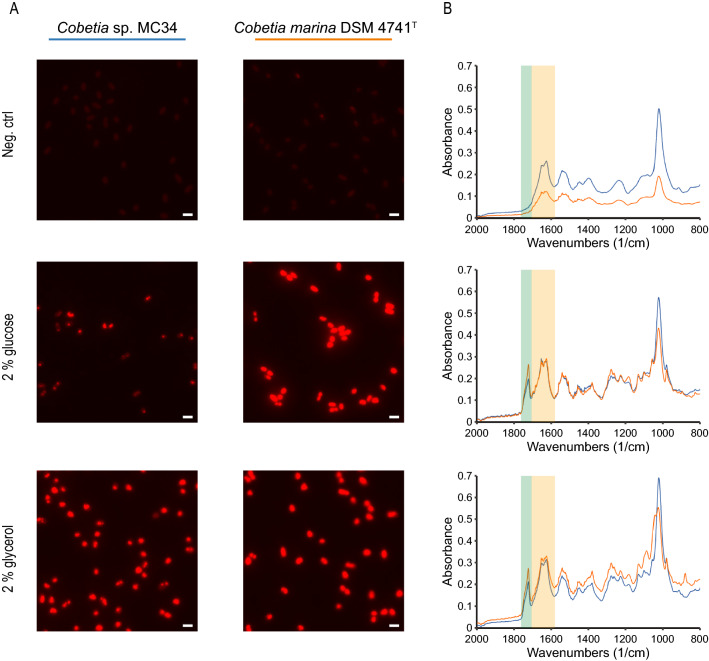
Putative PHA production on MMY agar plates supplemented with an excess carbon substrate. **A** Fluorescent microscopy of Nile-red stained cells grown on MMY agar plates supplemented with 2% glucose and 2% glycerol shows accumulation of intracellular inclusions after 72 h incubation at 14 °C, in contrast to the negative controls. The cells were imaged in a ZEISS Axio Observer 7 microscope at 100X magnification and the fluorescence intensity normalized to 1500 for all images. The scale bar shows 2 μm. **B** FT-IR spectra of cells from the same agar plates as in **A**. The cells grown on glucose and glycerol supplemented plates show carbonyl-ester signals centered at wavenumber 1724 cm^–1^ (Green box, n = 2). The amide band I-ester from proteins is observed in spectra from all plates, including the negative controls (Orange box, n = 2).

Putative PHA production for *Cobetia* sp. MC34 and *C. marina* DSM 4741^T^ was assessed after 72 h incubation at 14 °C on MMY agar plates supplemented with 2% glucose or 2% glycerol by FT-IR and fluorescent microscopy of cells stained with the lipophilic dye Nile-red. As shown in Fig. 2, cells from both strains grown on MMY agar supplemented with 2% glucose and 2% glycerol are short rods with strong intracellular fluorescent staining, typical for cells that produce PHA or other types of storage lipids [79, 80]. Cells grown without an additional carbon substrate showed little fluorescence. FT-IR analysis provides spectral information from vibration of molecules and can identify chemical functional groups even in a complex biological matrix. The carbonyl-ester group is particularly important for identification of PHA and gives a characteristic peak around wavelength 1725 cm^−1^ for crystalline PHB [81]. As seen in Fig. 2, the spectra of *Cobetia* sp. MC34 and *C. marina* DSM 4741^T^ grown on MMY agar supplemented with glucose or glycerol show carbonyl-ester peaks at 1723–1724 cm^−1^, in contrast to cells grown on the MMY control plates, where this peak is absent. These results suggests that PHA or other types of lipids are produced by the strains when supplemented these carbon sources.

### *Cobetia* sp. MC34 and *C. marina* DSM 4741^T^ exhibit mesophilic growth patterns with high production of PHB from acetate

**Fig. 3:**
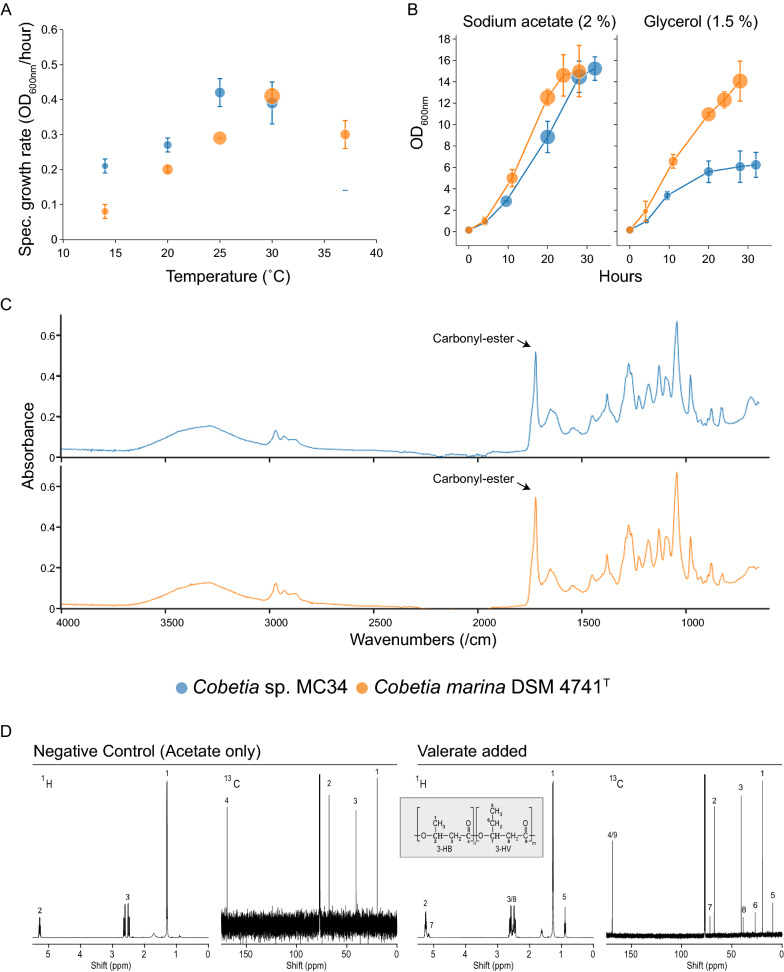
*Cobetia* sp. MC34 and *Cobetia marina* DSM 4741^T^ are mesophilic and produce PHB and PHBV. **A** *Cobetia* sp. MC34 (blue) and *C. marina* DSM 4741^T^ (orange) showed highest growth rate and putative PHA production per hour measured by FT-IR (visualized by sphere area) at 30 °C in 5 mL MMCY media supplemented with 2% glucose. The error bars show coefficient of variation of the specific growth rate between duplicate experiments (n = 6). **B** Growth curves in 80 mL MMCY and MMCY_2 media supplemented with 2% sodium acetate and 1.5% glycerol, respectively, at 30 °C, 200 rpm. The putative PHA production was measured by FT-IR (visualized by sphere area). The error bars show the coefficient of variation of the OD_600_ between duplicate experiments (n = 6). **C** FT-IR spectra of cells grown in 80 mL MMCY_2 media supplemented with 2% sodium acetate at 30 °C, 200 rpm for 28 h (*C. marina* DSM 4741^T^) and 32 h (*Cobetia* sp. MC34). The carbonyl-ester peaks at wavenumber 1724 cm^−1^ (arrows) indicate PHA production, and high amounts of PHB was confirmed by GC-FID analysis. **D** NMR confirms PHBV production in *Cobetia* sp. MC34. When grown on MMCY_2 media supplemented with 20 g/L sodium acetate, 3-HB were produced as indicated by peaks 1, 2 and 3 in the proton ^1^H plot (negative control) and peaks 1, 2, 3 and 4 in the carbon ^13^C plot (negative control). Co-supplement with 8 mM sodium valerate resulted in peaks unique to 3-HV as indicated by 5, 7, and 8 in the ^1^H plot and 5, 6, 7, 8, and 9 in the ^13^C plot. The spectra were normalized to the non-solvent peak with the highest intensity

Growth and putative PHA production were further investigated in small scale liquid cultures in MMCY media at different temperatures. *Cobetia* sp. MC34 and *C. marina* DSM 4741^T^ both exhibit growth patterns characteristic of mesophilic bacteria with the highest specific growth rate (~ 0.4 OD_600_/h) observed at 25–30 °C for *Cobetia* sp. MC34 and at 30 °C for *C. marina* DSM 4741^T^ (Fig. 3A). A higher specific growth rate for *Cobetia* sp. MC34 compared to *C. marina* DSM 4741^T^ was observed at 25 °C and below, and neither of the strains showed any significant growth at 6 °C. Good growth was observed for *C. marina* DSM 4741^T^ at 37 °C, although not optimal as expected based on the type strain description [27]. This may be due to differences in growth media or other conditions. In contrast, *Cobetia* sp. MC34 showed poor growth at 37 °C.

The putative PHA production was measured by FT-IR and calculated as the increase in the ratio between the carbonyl-ester region relative to the amide band I-ester region per hour after reaching stationary phase (C:A 1 ratio/h). The highest putative PHA production per hour was observed at 30 °C for both strains (Fig. 3A). *Cobetia marina* DSM 4741^T^ showed a higher putative PHA production than *Cobetia* sp. MC34 at all tested temperatures, despite the lower specific growth rate observed at temperatures below 25 °C. The sodium requirement and tolerance to salt in the MMCY media at 30 °C using glucose as carbon source was found to be in the range of 0–10% sodium chloride for *Cobetia* sp. MC34, with highest specific growth rates obtained within the range of 1–4% sodium chloride (Additional file 1: Fig. S1). Both strains thus classify as halotolerants when grown in MMCY media [82].

PHA production was subsequently determined and characterized after growth in shake-flask cultures at 30 °C, using MMCY and MMCY_2 media supplemented with either 2% sodium acetate, 1.5% glycerol, 4% glucose, or 4% fructose. Samples harvested along the growth curve were analyzed by FT-IR to monitor changes in the C:A 1 ratio in near-real time to determine when to harvest samples for GC-FID analysis. Supplement with acetate resulted in the highest OD_600_ and highest C:A 1 ratio for *Cobetia* sp. MC34 while acetate and glycerol were equally good for *C. marina* DSM 4741^T^ (Fig. 3B and C). Lower maximum OD_600_ values and lower C:A 1 ratios were found for *Cobetia* sp. MC34 when glycerol (Fig. 3B), glucose, and fructose were supplemented (Additional file 1: Figs. S2 and S3). An increase in the C:A 1 ratio was, however, still observed as the OD_600_ increased (Additional file 1: Fig. S3). The FT-IR analyses further showed highest intensity of the carbonyl-ester peak at 1724 ± 2 cm^–1^ for all conditions, similar to the FT-IR spectra obtained from cells grown on MMY agar and in small scale cultures. Additionally, peaks corresponding to CH_3_, CH_2_, CH_3_/CH_2_, and C=O-ester groups characteristic of lipids such as from fatty acids and polyesters were detected in all FT-IR spectra [83].

Cells harvested at the end of these growth experiments were lyophilized and subjected to GC-FID analysis for quantification of PHA. As shown in Table 1, the highest production of PHB from the use of a single substrate was found in cells supplemented with acetate, where 72 ± 11% and 61 ± 8.3% PHB/CDW were produced for *Cobetia* sp. MC34 and *C. marina* DSM 4741^T^, respectively*.* Production of PHB was also detected for both strains when glycerol, glucose, and fructose were used as carbon substrates.

**Table 1:** PHA productivity for *Cobetia* sp. MC34 and *C. marina* DSM 4741^T^

Strain	Carbon substrate	OD_600_ at harvest	CDW (g/L)	PHB (% of CDW)	PHV (% of CDW)	PHA (g/L)
*Cobetia* sp. MC34	Acetate	15.0 (± 1.3)	3.4 (± 0.5)	72 (± 11)	–	2.5
	Acetate + valerate	14.2 (± 2.1)	3.4 (± 0.8)	48 (± 3.4)	14 (± 2.3)	2.1
	Glycerol	6.2 (± 1.4)	2.5 (± 0.5)	26 (± 12)	–	0.7
	Glucose	6.5 (± 0.3)	1.8 (± 0.1)	35 (± 4.8)	–	0.6
	Fructose	6.0 (± 0.8)	3.9 (± 0.8)	9.9 (± 1.3)	–	0.4
*Cobetia marina* DSM 4741^T^	Acetate	15.0 (± 2.9)	3.9 (± 0.6)	61 (± 8.3)	–	2.4
	Acetate + valerate	17.0 (± 1.4)	4.4 (± 0.3)	59 (± 11)	26 (± 2.6)	3.7
	Glycerol	14.0 (± 2.1)	4.0 (± 0.1)	61 (± 8.1)	–	2.5
	Glucose	8.0 (± 0.2)	2.4 (± 0.3)	46 (± 2.9)	–	1.1
	Fructose	8.1 (± 0.2)	3.1 (± 0.2)	28 (± 11)	–	0.9

The cells were harvested from 80 mL cultures in MMCY_2 medium supplemented with 2% sodium acetate with- and without 8 mM sodium valerate, with 1.5% glycerol, 4.0% glucose or 4.0% fructose and were quantified by GC-FID analysis (n = 6). All *Cobetia sp*. MC34 cultures were grown for 32 h. The *C. marina* DSM 4741^T^ cultures supplemented with glucose and fructose were grown for 24 h, and those supplemented with acetate, valerate and glycerol were grown for 28 h. Values in parentheses are standard deviations calculated from 6 samples.

*Cobetia marina* DSM 4741^T^ produced more PHA than *Cobetia* sp. MC34 when glycerol and fructose were supplemented, with glycerol yielding 61% PHB/CDW, corresponding to a productivity of 2.5 g PHB/L similar to the productivity when acetate was supplemented. On the other hand, *Cobetia* sp. MC34 produced 26% PHB/CDW corresponding to 0.7 g PHB/L when fed with glycerol. Supplement with fructose resulted in high accumulation of biomass but only 9.9% PHB/CDW for *Cobetia* sp. MC34 while glucose supplementation resulted in low biomass production but accumulation of 35% PHB/CDW and corresponding productivity values of 0.4 and 0.6 g PHB/L, respectively. This is in contrast to *C. marina* DSM 4741^T^ that produced approximately 46% and 28% PHB/CDW from glucose and fructose, corresponding to a productivity of 1.1 and 0.9 g PHB/L. Substrates such as sucrose and *N*-acetylglucosamine were also tested but resulted in limited growth and no PHA production (data not shown).

### PHBV produced by *Cobetia* sp. MC34 and *C. marina* DSM 4741^T^ from acetate co-supplemented with valerate

The ability to produce co-polymers was tested by co-supplementing acetate (C_2_) cultures with a second carboxylic acid (C_5_, C_6_, and C_8_) at a C_2_:C_5/6/8_ ratio of 31 to 1. Both strains produced the co-polymer PHBV only when valerate (C_5_) was added as a co-substrate. Highest PHA productivity and a high proportion of the C_5_ monomer 3-hydroxyvalerate (3-HV) was found when *C. marina* DSM 4741^T^ was co-fed with acetate and valerate. The contents of 3-hydroxyalkanoate monomers, expressed as percentage of the CDW, was 59 ± 11% of 3-hydroxybutyrate (3-HB) and 26 ± 2.6% of 3-HV in the co-polymer produced by *C. marina* DSM 4741^T^, while 48 ± 3.4% of 3-HB and 14 ± 2.3% of 3-HV constituted the CDW of *Cobetia* sp. MC34 (Table 1). The compositions of PHB and PHBV extracted from *Cobetia* sp. MC34 were additionally confirmed by proton ^1^H and carbon ^13^C NMR analysis. As indicated in Fig. 3D, proton signals at 0.89 ppm (d, 3H) from the methyl group (1) of 3-HB are visible in both the negative control spectrum and the spectrum co-supplemented with valerate, whereas an additional methyl proton signal (5) at 1.27 ppm (t, 3H) is seen for 3-HV. The methylene protons from the main chains of 3-HB and 3-HV (3/8) are visible at 2.55 ppm (m, 4H). The methine protons for 3-HB and 3-HV (2/7) are visible at 5.25 and 5.16 ppm (m, 1H), respectively. A unique signal for the 3-HV methylene side chain (6) is visible at 5.25 ppm (m, 2H). The presence of 3-HV is also confirmed by ^13^C NMR by four peaks for 3-HV, which are not present in the negative control: The side chain 3-HV methyl carbon (5) at 9.4 ppm, the side chain methylene carbon (6) at 26.9 ppm, the main chain methylene carbon (8) at 38.8 ppm and the methine carbon (7) at 71.9 ppm [84]. Co-supplement with sodium salts of the mcl-precursors hexanoate and octanoate did not result in production of PHA co-polymers (data not shown).

### The genetic background for PHA production from acetate

**Fig. 4:**
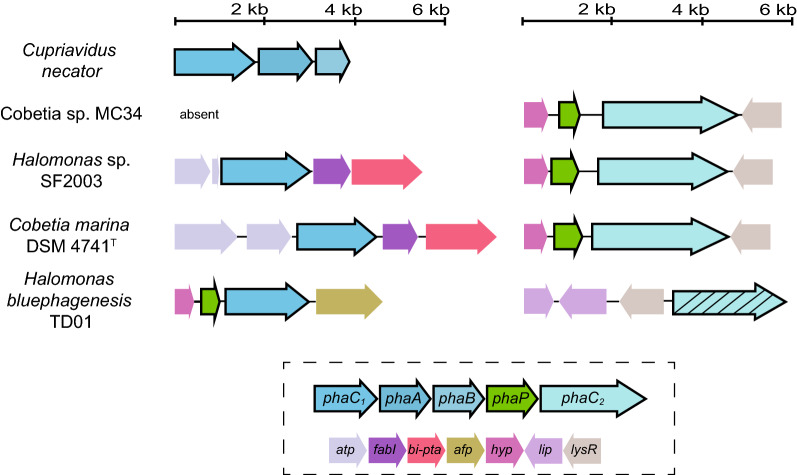
The *phaC*_2_ is the only PhaC polymerase gene in *Cobetia* sp. MC34. Gene synteny for the up-and downstream region of the PHA polymerase genes *phaC*_1_ and *phaC*_2_ found in selected *Cobetia* species. The *phaCAB* operon in *C. necator* is included for reference. The gene synteny of the *phaC*_2_ region is shared between *Cobetia* sp. MC34, *Halomonas* sp. SF2003 and *Cobetia marina* DSM 4741^T^. A putative non-homologues *phaC*_2_ variant (striped symbol) is also found in *H. bluephagenesis* TD01 but with different genes located in the upstream region. Gene abbreviations: *phaC*_1*/*2_ Poly(3-hydroxyalkanoate) polymerase subunit PhaC, PHA polymerase (EC 2.3.1.-), *phaA* Acetyl-CoA acetyltransferase (EC 2.3.1.9), *phaB* Acetoacetyl-CoA reductase (EC 1.1.1.36), *phaP* Phasin, *atp* ATP-metabolism genes, *fabI* (Enoyl-[acyl-carrier-protein] reductase [NADH], *bi-pta* bifunctional enoyl-CoA hydratase/phosphate acetyltransferase, *afp* Aminohydrolase family protein, *hyp* hypothetical protein of unknown function, *lip* lipoyl synthase and transferase, *lysR* LysR family transcriptional regulator

The finding that both *Cobetia* sp. MC34 and *C. marina* DSM 4741^T^ are good producers of PHB and PHBV prompted further investigation of their PHA biosynthesis genes. The genes responsible for PHA production are organized in a characteristic *phaCAB* operon in the model bacterium *C. necator* [85]*,* whereas the *phaA*, *phaB*, and *phaC* genes are typically located at independent loci in members of *Halomonas* [86, 87]. Similarly, the annotation of *Cobetia* sp. MC34 identified these three genes located in separate loci (Table 2). Interestingly, *Halomonas* sp. SF2003 and *C. marina* DSM 4741^T^ encode two copies of *phaC* (*phaC*_1_ and *phaC*_2_) that differ in length [75, 76] (Table 2). However, only one *phaC* gene was found in *Cobetia* sp. MC34 and named *phaC*_2_ (IZU87_04860) based on similarity to *phaC*_2_ from *Halomonas* sp. SF2003 (Table 2). A comparative analysis of the *phaC*_2_ gene region in *Cobetia* sp. MC34, *C. marina* DSM 4741^T^ and *Halomonas* sp. SF2003 showed conservation and similar gene synteny (Fig. 4). Located upstream of *phaC*_2_ is *phaP* (IZU87_04855) that encodes a phasin and a gene of unknown function *hyp* (IZU87_04850). Downstream is a LysR family transcriptional regulator gene *lysR* (IZU87_04865) that is conserved between all three *Cobetia* strains. This latter gene is also located upstream of the putative *phaC*_2_ found in *H. bluephagenesis* TD01 [86]. The gene synteny downstream of *phaC*_1_ is conserved between *Halomonas* sp. SF2003 and *C. marina* DSM 4741^T^ and consist of the gene enoyl-[acyl-carrier protein] reductase I *fabI* (IZU87_11845) and a gene annotated as a bifunctional enoyl-CoA hydratase/phosphate acetyltransferase *bi-pta* (Fig. 4).

**Table 2:** Genes encoding transporters, enzymes, transcriptional regulators and carbonosome proteins involved in PHA synthesis from acetate

Gene description	Gene(s)	*Cobetia* sp. MC34	*Cobetia marina* DSM 4741^T^	*Halomonas* sp. SF2003
Acetyl-CoA transferase (EC 2.3.1.9)	*phaA (phaA/fadA)*	+ [+] IZU87_07675/(IZU87_02125)	++ BFX80_16410/(BFX80_14370)	++ C8233_08335/C8233_10360
Acetoacetyl-CoA reductase (EC 1.1.1.36)	*phaB*	+ IZU87_12765	+ BFX80_01220	+ C8233_12530
Polyhydroxyalkanoate polymerase (EC 2.3.1.-)	*phaC*_1_/*pha*C_2_	−/+ −/IZU87_04860	+/+ BFX80_07630/BFX80_12110	+/+ C8233_00780/C8233_06040
Phosphate acetyltransferase (EC 2.3.1.8)	*pta*	+ IZU87_07815	+ BFX80_04540	+ C8233_15920
Bifunctional enoyl-CoA hydratase/phosphate acetyltransferase	*bi-pta*	–	+ BFX80_07620	+ C8233_00770
Acetate/propionate kinase (EC 2.7.2.1)	*ackA*	–	+ BFX80_07615	+ C8233_00765
Acetate–CoA ligase (EC 6.2.1.1)	*acs*	+ IZU87_06405	+ BFX80_02550	+ C8233_13850
Propionyl-CoA synthetase (EC 6.2.1.17)	*prpE*	+ IZU87_15385*	+ BFX80_09760	+ C8233_03035
Citrate synthase (EC 2.3.3.1)	*gltA*	+ IZU87_01890	+ BFX80_14605	+ C8233_08570
Aconitate hydratase (EC 4.2.1.3)	*acnA/acnB*	+/+ IZU87_02425/IZU87_05790	+/+ BFX80_01545/BFX80_04820	+/+ C8233_12860/C8233_16190
Isocitrate lyase (EC 4.1.3.1)	*AceA*	+ IZU87_14450	+ BFX80_03560	+ C8233_14950
Malate synthase (EC 2.3.3.9)	*AceB (glcB)*	+ IZU87_07285	+ BFX80_16805	+ C8233_10755
Cation acetate symporter	–	+ IZU87_06430	+ BFX80_02575	+ C8233_13875
Phasin	*phaP*_1_*/P*_2_	+/+ IZU87_08295/IZU87_04855	+/+ BFX80_04055/BFX80_12115	+/+ C8233_15445/C8233_06045
Repressor	*phaR*	+ IZU87_03745	+ BFX80_15490	+ C8233_09450
3-hydroxybutyrate dehydrogenase (EC 1.1.1.30)	*bdhA*	+ IZU87_16460	+ BFX80_07905	+ C8233_01125
Oxoacid transferases (EC 2.8.3)	*scoA/scoB*	+/+ IZU87_09115/IZU87_09110	+/+ BFX80_11345/BFX80_11350	+/+ C8233_05175/C8233_05180

The genes were identified from genome annotations [75, 76] and BLAST search against the different genomes

+ indicates presence and number of gene(s), with locus tags, − indicate absence, [+] indicate ambiguity between the annotation methods Prokka [57] and NCBI’s Prokaryotic Annotation Pipeline [58]

*Annotated as acetate–CoA ligase (EC 6.2.1.1) by NCBI’s Prokaryotic Annotation Pipeline [58], but as propionyl-CoA synthetase (EC 6.2.1.17) by KEGG [67]

An acetate kinase gene *ackA* is furthermore found downstream of *fabI* and *bi-pta* in both *C. marina* DSM 4741^T^ and *Halomonas* sp. SF2003 (not shown). A similar gene synteny for *fabI*, *bi-pta*, and *ackA* is found in *H. bluephagenesis* TD01 (not shown), but with the difference that *phaC*_1_ is located at a separate loci (Fig. 4). The gene *fabI* (IZU87_11845) is present in the genome of *Cobetia* sp. MC34, but the *ackA* and *bi-pta* genes were not found. This suggests that *phaC*_1_ could have been lost during evolution. The possibility that the absence of *phaC*_1_ in *Cobetia* sp. MC34 resulted from a genome assembly artefact was considered and evaluated by investigation of *phaC* variants found in other members of the *Cobetia* genus. Two copies of *phaC* are found in 8 out of 19 whole genome sequences and we consider it unlikely that assembly artefacts are accountable for the presence of only a single copy in the remaining eight genomes.

Two routes to convert propanoate (originating from valerate) to propanoyl-CoA were found in *Cobetia* sp. MC34: The acetate–CoA ligase Acs (*acs,* IZU87_06405) and the propionyl-CoA synthetase PrpE (*prpE*, IZY87_15385), both of which convert propanoate to propanoyl-CoA via a propionyl-adenylate intermediate [67]. The *prpE* (IZU87_15385) was annotated as one of two *acs* genes by NCBI’s Prokaryotic Annotation Pipeline [58], but KEGG annotation [67] and its high amino acid sequence similarity to PrpE in *C. marina* DSM 4741^T^ and *Halomonas* sp. SF2003 (Additional file 1: Table S2) suggest *prpE* to be the most correct gene annotation (Table 2). The AckA-Pta found in *C. marina* DSM 4741^T^ and *Halomonas* sp. SF2003 constitute a third route for generating propanoyl-CoA from propanoate but via a propanoyl phosphate intermediate [67]. The Acs and Pta-AckA are furthermore responsible for conversion of acetate to acetyl-CoA and therefore likely to be important determinants for acetate turnover into biomass and PHA in *Cobetia* sp. MC34 and *C. marina* DSM 4741^T^ [67]. Both strains obtained high PHA production, cell densities (OD_600_), and high biomass accumulation when supplemented with acetate. These results might therefore be further explained by enzymes belonging to the glyoxylate shunt pathway. In essence, the glyoxylate shunt function as a shortcut in the TCA cycle where it starts with conversion of acetyl-CoA to citrate by the citrate synthase GltA (*gltA,* IZU87_01890) and via isocitrate by aconitate hydratases AcnA/B (*acnA/B,* IZU87_02425/IZU87_05790) to glyoxylate by isocitrate lyase AceA (*aceA,* IZU87_14450), before malate is formed by addition of an acetyl-CoA molecule by AceB (*aceB,* IZU87_07285) [88]. In contrast to the TCA cycle, the glyoxylate shunt does not lead to loss of CO_2_ and requires fewer enzymatic steps to generate malate, the precursor for biomass production [89]. In addition, a putative cation acetate symporter (IZU87_06430) was found. Taken together, these genes are likely important for explaining the good growth and high PHA production achieved using acetate as carbon source for both *Cobetia* sp. MC34 and *C. marina* DSM 4741^T^ (Table 2).

Of note, genome annotation by Prokka [57] identified the gene *fadA* (IZU87_02125) as a second *phaA* copy in *Cobetia* sp. MC34. The amino acid sequence of this gene share 95.7% similarity with the PhaA annotated in *C. marina* DSM 4741^T^ and 42.4% to the PhaA annotated in *Halomonas* sp. SF2003 (Additional file 1: Table S2). We thus consider the function of this gene to be ambiguous. The exact copy number of *phaA* and *phaB* remains elusive since no experimental evidence concerning these has been presented in *Cobetia*. As also shown in Additional file 1: Table S2, the highest percentage of amino acid identity was found between homologs of *Cobetia* sp. MC34 and *Halomonas* sp. SF2003, which is in agreement with the phylogenies presented in Fig. 1.

### Other insights from genome analysis of *Cobetia* sp. MC34 related to PHA metabolism

A closer look at the genomes of *Cobetia* sp. MC34, *Halomonas* sp. SF2003 and *C. marina* DSM 4741^T^ gave additional insights in regard to PHA metabolism (Table 2). Copies of the PHA synthesis repressor gene *phaR* (IZU87_03745) and a second phasin gene, *phaP*_1_ (IZU87_08295), were found at independent loci without proximity to any known PHA related genes. The depolymerase PhaZ is important for the biological turnover of PHA and potentially for use in processing of PHA bioplastic waste by enzymatic degradation similar to biorecycling by enzymatic degradation of polyethylene terephthalate (PET) by PET hydrolases [90]. Surprisingly, a *phaZ* gene was not present in *Cobetia* sp. MC34, in *Halomonas* sp. SF2003, nor in *C. marina* DSM 4741^T^ (Table 2). However, the 3-hydroxybutyrate dehydrogenase BdhA (*bdhA,* IZU87_16460) which may interconvert the PhaZ hydroxyacyl product (e.g., (R)-3-hydroxybutanoic acid) to an oxoacid (e.g., acetoacetate) was found in all three strains (Table 2). Also, the ScoA/B (*scoA/B,* IZU87_09115/IZU87_09110) responsible for converting the oxoacid to oxoacyl-CoA (*e.g.*, acetoacetyl-CoA, the substrate for PhaB) were present in all (Table 2). This indicates the existence of one or more yet unidentified PhaZ depolymerases located upstream in the PHA biodegradation pathway. Also, additional BLAST search using PhaZ sequences from *P. oleovorans* and *H. bluephagenesis* TD01 against the *Cobetia* sp. MC34 genome did not find any clear PhaZ matches. Several putative genes involved in fatty acid biosynthesis and fatty acid degradation (ß-oxidation) were found in the genome with a high degree of redundancy, as expected (Additional file 1: Table S3). These genes can have redundant functions in PHA metabolism and are important targets for future studies, e.g., if mcl-PHA production is attempted by genetic engineering techniques.

### *Cobetia* PhaC_2_ is a class I PhaC polymerase that contains an extended C-terminus with a putative role in DNA-binding

The finding that all whole genome sequences of *Cobetia* encode one or two PhaCs led us to study them in more detail. PhaC_1_ and PhaC_2_ from *Halomonas* sp. SF2003 are experimentally confirmed to belong to class I PhaC polymerases [87, 91]. The molecular weight of *Halomonas* sp. SF2003 PhaC_1_ is calculated to be 71.5 kDa, which is close to the molecular weight for other class I PhaC polymerases such as in *C. necator *[20]*.* A homolog of the *phaC*_2_ gene in *Halomonas* sp. SF2003 was found in all *Cobetia* genomes and is the only *phaC* gene present in eleven out of nineteen genomes, including in *Cobetia* sp. MC34*.* The estimated sizes of the PhaC_2_ polymerases differ considerably from PhaC_1_ with predicted molecular weights ranging from 93.6 to 106.4 kDa (Table 3). All PhaC polymerases contain a PhaC box sequence (G/S)XCX(G/A)G with similarity to the lipase box motif found in lipases and PhaZ depolymerases [92]. Most important is the conserved cysteine residue in the center of the box, which constitutes the active center of the enzyme, while the X’s denounce non-conserved amino acids (AA). The PhaC boxes in *Halomonas* are described to contain either an SYCLG or GYCLG motif, while a GFCVGG motif is found in *C. necator* PhaC_1_ [86, 87, 91, 92]. All *Cobetia* PhaC_1_ and PhaC_2_ sequences analyzed in this study contained a GYCLG or SYCIG motif, respectively (Table 3).

**Table 3:** Characteristics of PhaC polymerases found in *Cobetia* and *Halomonas*

Strain	PhaC_2_ MW (kDa)/pI_N613_/pI_C-terminus_	PhaC_2_ box	HC2 domain (E-value)	PhaC_1_ MW (kDa)/pI	PhaC_1_ box
*Cobetia* sp. MC34	105.4/4.63/10.34	SYCIG	8.77 × 10^–9^	–	–
*Cobetia amphilecti* KMM296	103.1/4.67/10.27	SYCIG	4.56 × 10^–9^	–	–
*Halomonas* sp. SF2003*	101.8/4.66/10.15	SYCIG	4.79 × 10^–10^	71.5/5.27	GYCLG
*Halomonas* sp. GDM18*	102.6/4.63/10.24	SYCIG	2.99 × 10^–8^	71.5/5.27	GYCLG
*Cobetia* sp. AM6	102.6/4.63/10.34	SYCIG	3.55 × 10^–9^	71.5/5.31	GYCLG
*Cobetia* sp. UCD-24C	103.9/4.76/10.19	SYCIG	2.24 × 10^–8^	–	–
*Cobetia* sp. QF1	95.1/4.77/10.23	SYCIG	3.79 × 10^–11^	–	–
*Cobetia* sp. ICG0124	106.1/4.74/10.23	SYCIG	4.32 × 10^–12^	40–50 kDa*	GYCLG
*Cobetia* sp. L2A1	99.9/4.85/10.06	SYCIG	1.86 × 10^–7^	65.6/5.43	GYCLG
*Cobetia crustatorum* SM1923	93.6/4.77/10.14	SYCIG	2.78 × 10^–10^	–	–
*Cobetia crustatorum* JO1	95.4/4.77/10.23	SYCIG	3.42 × 10^–11^	–	–
*Cobetia marina* DSM 4741^T^	106.4/4.74/10.35	SYCIG	1.77 × 10^–10^	64.0/5.31	GYCLG
*Cobetia marina* NBRC 15607	105.2/4.74/10.36	SYCIG	2.85 × 10^–10^	63.9/5.31	GYCLG
*Cobetia marina* T1	105.1/4.74/10.36	SYCIG	2.73 × 10^–11^	64.1/5.26	GYCLG
*Cobetia* sp. MM1IDA2H-1	105.0/4.74/10.40	SYCIG	3.39 × 10^–7^	–	–
*Cobetia pacifica* GPM2	105.9/4.74/10.38	SYCIG	1.14 × 10^–9^	–	–
*Cobetia sp. IU 180733JP01* *(5-11-6-3)*	100.9/4.74/10.36	SYCIG	3.04 × 10^–9^	–	–
*Cobetia sp. 190790JP01* *(5-25-4-2)*	106.2/4.74/10.38	SYCIG	3.09 × 10^–9^	–	–
*Cobetia* sp. MB87	100.4/4.66/10.22	SYCIG	2.91 × 10^–10^	–	–
*Halomonas* sp. R5-57	71.8/4.86/8.54**	SYCVG	–	66.7/5.29	GYCLG
*Halomonas bluephagenesis* TD01	92.8/5.39/9.60***	GNCQAG	–	69.7/4.80	SYCVG
*Halomonas* sp. O-1	89.0/5.45/10.10***	GNCQAG	–	69.4/5.18	SYCVG

PhaC_1_ and PhaC_2_ sequences found in *Cobetia* whole genome sequences from genome annotation and by BLAST search. Predicted Molecular weight (MW) is shown for the complete protein sequence while the isoelectric point (pI) is shown for the N-terminal 613 amino acids (pI_N613)_ and for the extended C-terminus (pI_C-terminus_) for PhaC_2_ and for the whole protein sequence for PhaC_1_.Genome accession numbers are listed in Additional file 1: Table S1

*Identified as belonging to *Cobetia* (this study)

**The C-terminus consist of only 21 amino acids and thus non-homologous to *Cobetia* PhaC_2_

***Putative PhaC_2,_ non-homologous to *Cobetia* PhaC_2_ (This study)

Further analyses of the *Cobetia* sp. MC34 PhaC_2_ (993 AA) reveal high amino acid similarity to putative homologs in *Halomonas* sp. GDM18 (962 AA, 94.7% identity), *C. amphilecti* (967 AA, 94.6% identity), *Halomonas* sp. SF2003 (954 AA, 93.7% identity), and *Cobetia* sp. AM6 (961 AA, 93.4% identity). Protein BLAST also revealed high sequence similarity for amino acid positions 76–613 to the PHA_synth_I domain (NCBI CDD TIGR01838) in PhaC_1_ of *C. necator*, while no domain was identified for the remaining C-terminus similar to previous analysis of *Halomonas* sp. SF2003 PhaC_2_ [76]. However, when querying *Cobetia* sp. MC34 PhaC_2_ against NCBI’s Conserved Domain Database (NCBI CDD) using less stringent search criteria, many unspecific hits with relatively low e-values were detected. Among these were the “Histone H1-like nucleoprotein HC2 domain” (HC2 Domain, NCBI CDD pfam07382, E-value 8.77 × 10^–9^), the “transcriptional regulator ICP4 domain” (NCBI CDD PHA03307, E-value 0.25 × 10^–13^), and the “DNA polymerase III subunit gamma/tau domain” (NCBI CDD PRK07003, E-value 5.20 × 10^–11^). Investigation of all 19 *Cobetia* PhaC_2_ sequences identified similar non-specific hits to the “HC2 domain” in the extended C-terminus in all *Cobetia* PhaC_2_ sequences (Table 3). The “HC2 domain” was, however, not the only hit with a low E-value identified in the extended C-terminus of the *Cobetia* PhaC_2_ sequences. The domain “Herpes_BLLF1” (NCBI CDD pfam05109) was found in all, and “Endomucin” (NCBI CDD pfam07010) was found in all except *C. crustatorum* SM1923 PhaC_2_, in addition to many other non-specific hits found in most but not all PhaC_2_ C-termini (Additional file 2: S1).

Furthermore, comparison of the isoelectric point (pI) for the N-terminal 613 amino acids versus the extended C-terminus showed a marked difference of more than 5 pH units, with the C-terminus having a pI value of 10.34 for *Cobetia* sp. MC34 PhaC_2_, a pattern which was observed in all *Cobetia* PhaC_2_ sequences (Table 3). These high pI values for the C-termini indicate a possible role for binding negatively charged molecules such as the DNA backbone. The amino acid AAKP sequence motif is known to be prevalent in DNA-binding domains [93] and five such motifs were indeed identified in the extended C-terminus in *Cobetia* sp. L2A1 PhaC_2,_ two in PhaC_2_ of *Cobetia* sp. QF1, *C. crustatorum* JO1, and a single AAKP motif in *C. crustatorum* SM1923 PhaC_2_.

*Halomonas* sp. O-1 and *H. bluephagenesis* TD01 encode putative PhaC_2_ polymerases that contain “PhaC” domains (NCBI CDD COG3243) and therefore may be homologs to *Cobetia* PhaC_2_. However, their PhaC box motifs are different and PHA was not produced when *Halomonas* sp. O-1 PhaC_2_ was expressed in *E. coli* [86, 87]. The domain search did not identify a “HC2 domain” in any of the two. However, hits to “Predicted 5' DNA nuclease, H3TH domain” (NCBI CDD COG3743) and “DNA polymerase III subunits gamma and tau domain” (NCBI CDD PRK14951) were identified for *Halomonas* sp. O-1 PhaC_2_, while the “histone H1-like DNA-binding protein” (NCBI CDD NF038053) was identified in *H. bluephagenesis* TD01 PhaC_2_ and indicate DNA binding properties as well (Data not shown). Other PhaC variants include the PhaC_2_ from *Halomonas* sp. R5-57 that has a slightly different PhaC box and is closer in size to the *Cobetia* PhaC_1_ (Table 3).

**Fig. 5:**
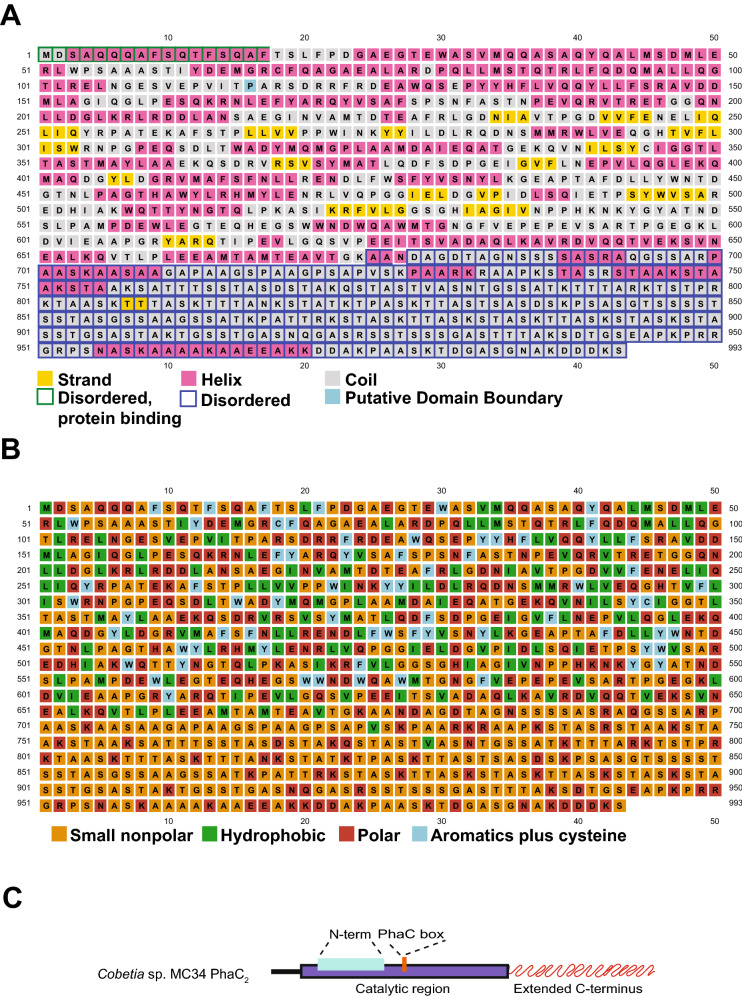
Secondary structure prediction of the PhaC_2_ polymerase in *Cobetia* sp. MC34. **A** The PSIPRED modeling plot predicts a high proportion of strand and helix secondary structures in the N-terminal (amino acids 1 to 674), whereas the extended C-terminus (amino acid 614 to 993) is predicted to consist primarily of unordered coiled domains, short helixes, and one strand domain. **B** The second PSIPRED plot shows an abundance of small non-polar and polar amino acids in the extended C-terminus including the positively charged lysine and arginine residues. **C** A generalized structure of the *Cobetia* sp. MC34 PhaC_2_ protein. The catalytic region is homologous to class I PhaC polymerases and contains an N-terminal PHA_synth_I domain and a SYCIG l PhaC box. The extended C-terminus (380 amino acids) has an unordered structure with a computed pI of 10.34.

Limited tertiary and quaternary structural information is available for PhaC polymerases with only two crystal structures of the catalytic domain currently published, the PhaC_1_ from *C. necator* [94, 95] and the PhaC_1_ from *Chromobacterium* sp. USM2, with- and without co-enzyme A [96, 97]. We therefore performed in silico secondary structure predictions by PSIPRED [68, 69] to shed more light on the function of the extended C-terminus of *Cobetia* sp. MC34 PhaC_2_. The PSIPRED analysis showed presence of several strand, helix, and coiled structures in the N-terminal domain (amino acids 1 to 674). The extended C-terminus (amino acid position 614 to 993) consisted mostly of unordered coils, although ten helixes and a single strand domain were present (Fig. 5A). Further, the C-terminus domain was found to contain a high proportion of small non-polar and polar amino acids, including positively charged lysine and arginine residues, consistent with a high theoretical pI (Fig. 5B). A generalized structure of PhaC_2_ from *Cobetia* sp. MC34 is shown in Fig. 5C.

## Discussion

In this study, a new strain identified as *Cobetia* sp. MC34 isolated from a fish-landing facility in the Arctic part of Norway was investigated as a putative PHA producer. The strain showed closest relationship to *C. amphilecti* and *Halomonas* sp. SF2003 based on 16S rRNA and whole genome analysis by ANI, which suggest placement of *Cobetia* sp. MC34 as a strain of *C. amphilecti* according to current taxonomy [28]. The genus type strain *C. marina* DSM 4741^T^ included in our study for comparison was the first and only *Cobetia* specie described after a split from genus *Halomonas* in 2002 [27]. Since then, the number of species allocated to the *Cobetia* genus has increased to five, and to 143 species within the *Halomonas* genus [98, 99]. In addition, the introduction of whole genome sequencing of bacteria and meta-genomes has led to deposition of hundreds of new genomes belonging to the two genera in the NCBI genome database. As a result, allocation of new strains belonging to *Cobetia* or *Halomonas* is often confusing and the nomenclature is, at times, used interchangeably. This problem is addressed by the scientific community and new standards for description of the *Halomonadacea* family appear to be underway [100].

*Cobetia* sp. MC34 and *C. marina* DSM 4741^T^ were found to be mesophilic, halotolerant, and PHA producing bacteria. The ability to grow in media with low levels of salt for *Cobetia* sp. MC34 was somewhat unexpected, given the isolation from a cold marine-associated environment. *Cobetia* sp. MC34, did however, show a higher specific growth rate than *C. marina* DSM 4741^T^ in the lower range of the tested temperatures, while *C. marina* DSM 4741^T^ appeared to have a higher putative PHA production than *Cobetia* sp. MC34 at all temperatures tested, as well as a lower optimum growth temperature than previously reported [27]. The basic growth- and PHA production phenotypes of *Cobetia* sp. MC34 and *C. marina* DSM 4741^T^ were subsequently characterized after growth on the industrially relevant pure substrates acetate, glycerol, glucose and fructose. Acetate proved to be a particularly promising carbon source for PHA production in both strains, and *Cobetia* sp. MC34 was able to produce 72% PHB/CDW and *C. marina* DSM 4741^T^ 61% PHB/CDW on this substrate. Co-supplement of acetate with valerate further showed that the same strains were able to produce 14% PHV/CDW and 26% PHV/CDW, respectively, in addition to production of PHB (Table 1). The high natural production of PHB and PHBV when using acetate with- and without valerate in both *Cobetia* sp. MC34 and *C. marina* DSM 4741^T^ is interesting from a biotechnological perspective. Acetate is a relatively low-cost carboxylic acid with a global production exceeding 10 million tons per year primarily synthesized from chemical routes, but synthesis of acetate is also possible from green materials such as lignocellulose feedstock or by bacterial fermentation [101]. To our knowledge, our study is the first to show PHA production from acetate in the genus *Cobetia*. However, others have reported efficient PHA production from acetate both naturally and by heterologous expression of acetate metabolism genes in different bacteria. For example, *Halomonas boliviensis* is able to produce 88% PHB/CDW when supplemented with a mixture of acetate and butyrate under optimized conditions [100] and *H. bluephagenesis* TD01 can produce more than 89% PHB/CDW under oxygen limitation when supplemented with a combination of acetic acid and glucose [102]. The latter study also demonstrated increased PHA production by engineering of the NADH/NAD^+^ pathway [102]. Neither of the studies reported PHA production from use of acetate alone, making a direct comparison with our results difficult. The industrial “workhorse” for PHA production, *C. necator,* is an efficient producer of PHA from acetate with production of up to 72% PHA/CDW corresponding to 43 g PHA/L achieved in a 3-L bioreactor [103]. *Escherichia coli* is unable to produce PHA naturally due to lack of *pha* biosynthesis genes. However, metabolic engineering has enabled *E. coli* to produce up to 42% PHB/CDW, 35% PHBV/CDW or 58% poly(3-hydroxybutyrate-*co*-4-hydroxybutyrate)/CDW from acetate with- or without addition of propionate [104]. Strains of *Pseudomonas putida* can naturally produce 20–30% mcl-PHA/CDW from acetate, and overexpression of *acs* has been shown to increase the yields [105, 106]. The amounts obtained in *Pseudomonas* are low compared to our results with *Cobetia* sp. MC34 and *C. marina* DSM 4741^T^, but the production is interesting from an industrial perspective due to the material properties of mcl-PHA. Taken together, these studies imply that there is an industrial potential of using acetate and different bacteria for production of PHA. Glycerol, a by-product form the biodiesel industry, is another substrate of interest for green production of PHA. Our study show that *C. marina* DSM 4741^T^ produced 61% PHB/CDW from glycerol, significantly more than *Cobetia* sp. MC34 which produced only 26% PHB/CDW (Table 1). *Halomonas* sp. SF2003, the close relative to *Cobetia* sp. MC34, has to our knowledge not been investigated for PHA production on acetate nor glycerol. However, this strain is able to produce PHA in amounts comparable or even higher than *Cobetia* sp. MC34 from single carbohydrates such as glucose [91]. More important from an industrial perspective is the finding that *Halomonas* sp. SF2003 is able to produce approximately 78% PHA/CDW from fruit industry process water containing a mixture of fructose and glucose in addition to various ash constituents [36]. This amount is significantly higher than achieved from glucose and fructose in *Cobetia* sp. MC34.

It should be emphasized that the PHA productivities found in our and the above mentioned studies are still low compared to those achieved in high-density fermentation of a genetically engineered- and artificially selected strain of *H. bluephagenesis* TD01 where more than 71- and 56 g/L PHA were produced using glucose as substrate [107]. This demonstrates that further optimization and scale up is needed to make *Cobetia* sp. MC34 and *C. marina* DSM 4741^T^ into industrially competitive strains for PHA production.

The somewhat lower production of PHB from acetate but higher production of PHV when co-supplemented with valerate in *C. marina* DSM 4741^T^ compared to *Cobetia* sp. MC34 could be due to the lack of the acetate kinase *ackA* in the draft genome of *Cobetia* sp. MC34 (Table 2). The acetate kinase AckA and the phosphate acetyltransferase Pta in *C. marina* DSM 4741^T^ offers an additional route to formation of propanoyl-CoA from propanoate via a propanoyl-phosphate intermediate in addition to the Acs and PrpE routes (Table 2) [67]. This may explain why *C. marina* DSM 4741^T^ was found to produce a higher content of PHV than *Cobetia* sp. MC34 when co-supplemented with valerate, since propanoyl-CoA is the precursor converted to 3-ketovaleryl-CoA by PhaA, which can be further converted to (R)-3-hydroxyvaleryl-CoA by PhaB, and subsequently to PHBV by PhaC. The apparent lack of *ackA* in *Cobetia* sp. MC34 means that only the high-affinity irreversible Acs route is possible for converting acetate to acetyl-CoA, whereas *C. marina* DSM 4741^T^ can also use the low-affinity but reversible Pta-AckA route [108]. Thus, *C. marina* DSM 4741^T^ might redirect acetyl-CoA back to acetate whereas *Cobetia* sp. MC34 must redirect excess acetyl-CoA to other routes, such as PHB production. A closed genome sequence of *Cobetia* sp. MC34 in combination with transcriptomic data is needed to verify if the apparent lack of *ackA* can in fact explain the observed differences in PHA production between the two strains using acetate with- and without co-supplement with valerate. Another possible explanation for differences in PHA production, such as when using glycerol, may be due to different *phaC* copy numbers (Table 2). Having an additional PhaC variant may make *C. marina* DSM 4741^T^ more robust and versatile in terms of PHA production. Interestingly, the study of *Halomonas* sp. SF2003 indicates that PhaC_2_ is capable of producing PHA from a higher number of carbon substrates than PhaC_1_ [91]. It remains, however, to be shown if the *phaC* variants in our strains are expressed differently upon feeding with different substrates.

The genome analyses of *Cobetia* sp. MC34 and *C. marina* DSM 4741^T^ identified all genes required for PHA production in both strains and the class I PHA polymerase PhaC_2_ was found to contain a long (> 300 amino acids) extended C-terminus of unknown function. This combined with the finding that *phaC*_2_ is the only *phaC* gene present in *Cobetia* sp. MC34, as well as in eleven other *Cobetia* members, made it an interesting candidate for further in silico studies.

The PSIPRED modeling of the secondary structure of *Cobetia* sp. MC34 PhaC_2_ predicted that the C-terminal region consist mostly of unordered coils, without apparent structure. Coiled structures are found ubiquitously in protein structures of all domains of life and are attributed to various biological functions. Several functions are therefore possible for the *Cobetia* PhaC_2_ extended C-terminus such as oligo- or dimerization domain, activation- or repression domain, chromosome segregating factor, molecular ruler, or as a transcription factor like DNA-binding domain [109]. It is generally established that PhaC polymerases from model organisms function as homo- or hetero-dimers [20]. PhaC_2_ is expected to localize at the carbonosome surface together with phasins and other proteins, from where PHA is produced and regulated, e.g., via a PhaM-like mediated activation, as described for *C. necator* [110]. The nucleoid is suggested to serve as scaffold for initiation of PHA synthesis via PhaC-PhaM binding, which allows for the carbonosome to segregate into daughter cells during cell division [110, 111]. The importance of PhaM for subcellular localization of the carbonosome and its distribution to daughter cells in *C. necator* was most recently demonstrated at the single-cell level using *C. necator* PhaM expressed in *E. coli*, where the *E. coli* gain-of-function mutant exhibited similar “behavior” as observed in *C. necator* [112]. Thus, although a PhaM homolog is absent in *Cobetia* sp. MC34, the possibility for the coiled structure in PhaC_2_ to serve as a nucleoid anchor in replacement of PhaM may be speculated. The PHA synthesis repressor PhaR from *Paracoccus denitrificans* has also been found to contain a short N-terminal DNA-binding domain, although the high proportion of phasins in the carbonosome suggested that it is more likely to be the free form of PhaR that binds to DNA, rather than a PhaR-carbonosome complex [113]. Similar to *Halomonas* sp. SF2003 [76], genome analysis of *Cobetia* sp. MC34 and *C. marina* DSM 4741^T^ identified *phaR*. However, interaction of PhaR with the carbonosome and chromosome needs to be addressed by in vivo studies. A DNA binding mechanism for PhaC and the carbonosome is also described in bacteria more closely related to *Cobetia*, such as in *P. oleovorans*. Here, PhaF is responsible for repression of *phaC* and *phaI* and is found located on PHA granules [114]. PhaF contains AAKP motifs that are also found in alginate transcriptional regulators and in histones [93]. Interestingly, we identified four *Cobetia* PhaC_2_ sequences that contain between one and five AAKP motifs in the extended C-terminus. The possibility for the *Cobetia* PhaC_2_ extended C-terminus to function in DNA-binding was further substantiated by our domain search, which identified the “Histone H1-like nucleoprotein HC2 domain” (HC2 Domain, NCBI CDD pfam07382) in all *Cobetia* PhaC_2_ sequences. Hits to several other domains including “Herpes_BLLF1” (NCBI CDD pfam05109) and “Endomucin” (NCBI CDD pfam07010) which are described in NCBI CDD as types of membrane proteins makes a role in anchoring the *Cobetia* PhaC_2_ synthase to the granule surface a possibility as well. Based on these considerations and in silico data, we propose that the extended C-terminus found in *Cobetia* PhaC_2_ could be responsible for binding of the PHA carbonosome to the bacterial DNA.

The apparent lack of the PHA depolymerase gene *phaZ* in the genome of *Cobetia* sp. MC34 is comparable to recent findings from a consortium of marine bacteria capable of degrading PHA, where the *phaZ* gene was predicted in only 3 out of 33 strains from 7 genera (3). Annotations of *phaZ* in *Halomonas* and *Cobetia* additionally suffers from limited experimental validation as the three *phaZ* sequences annotated in *H. bluephagenesis* TD01 were not supported by non-PHA degrading phenotypes in knock-down experiments [115]. Identification of the *Halomonas* and *Cobetia* PhaZ depolymerases may enable research on biological recycling of PHA bioplastic and thus constitute important future biotechnological research targets. Both *Cobetia* sp. MC34 and *C. marina* DSM 4741^T^ are natural producers of PHA and potential candidates in the field of blue biotechnology with their salt- and alkali tolerance, mesophilic growth pattern and PHA production from green substrates. This may allow for upscaling experiments using standard fermentation equipment in order to increase biomass- and PHA production to industrially relevant titers. It remains, however, an open question whether megatons industrial scale production of PHA is economically and environmentally competitive with, e.g., chemical recycling of “oil plastics” produced from biological source material. Even if not, production of PHA monomers for use as green platform chemicals convertible into, e.g., crotonic acid [116] might prove just as important in a future green biorefinery that utilizes a spectrum of biological processes for substitution of fossil petroleum.

## Conclusion

*Cobetia* sp. MC34 and *Cobetia marina* DSM 4741^T^ are producers of scl-PHA from pure substrates obtainable from “green” sources, such as acetate. However, further optimization of growth conditions will be needed to increase biomass- and PHA production to industrially relevant titers. Optimization studies may also include genetic engineering. The observed difference in acetate metabolism genes as well as the presence of one or two copies of *phaC* in *Cobetia* warrant further studies to elucidate their impact on PHA biosynthesis. An interesting PhaC_2_ polymerase variant with an extended C-terminus was present in all *Cobetia* genomes investigated. Based on in silico analyses, we speculate that the *Cobetia* PhaC_2_ extended C-terminus is involved in DNA-binding.

## Supplementary Information

**Additional file 1: Figure S1.** Tolerance- and requirement to sodium chloride in MMCY media supplemented with 2 % glucose at 30 °C, 200 rpm, shows highest specific growth rate in the range of 1-4 % for both strains (n = 6). **Figure S2.** Representative raw FTIR spectra for *Cobetia *sp. MC34 (blue) and *Cobetia marina* DSM4741^T^ (orange) grown in MMCY and MMCY_2 media supplemented with 1.5 % glycerol, 4 % glucose, 4 % fructose or 20 g/l acetate + 8 mM sodium valerate (n = 1). **Figure S3.** Growth curves measured by optical density (OD600nm) for the strains *Cobetia* sp. MC34 (blue) and *Cobetia marina* DSM 4741^T^ (orange) grown in MMCY media supplemented with 40 g/l glucose or fructose, respectively. The carbonyl-ester to amide band I-ester ratio obtained by FTIR is visualized as sphere area and indicate putative PHA production, which was determined at the end of the experiment by chloroform extraction and GC/MS quantification. Error bars represent standard deviation of OD600nm (n = 6). **Table S1.** List of *Halomonadacea* sequences used to construct the 16S- and ANI trees, and information about the strains. **Table S2.** Amino acid sequence similarities of the transporters, enzymes, transcriptional regulators and carbonosome proteins involved in PHA synthesis from acetate. **Table S3.** Fatty acid biosynthesis (FAS) and fatty acid degradation (ß-oxidation) genes found in *Cobetia* sp. MC34, derived from the genome annotation.

**Additional file 2:** PhaC_C-term.

## Data Availability

The datasets used and/or analyzed in this study are available from the corresponding author on reasonable request. Genome data for *Cobetia* sp. MC34 is available from NCBI under accessions listed in the “*Methods*” section “*Genome sequencing and assembly*”.

## Abbreviations

AA: Amino acids
ANI: Average Nucleotide Identity
BLAST: Basic Local Alignment Search Tool
bp: Base pairs
CDW: Cell dry weight
FT-IR: Fourier transform infrared spectroscopy
GC-FID: Gas Chromatography-Flame Ionization Detector
KEGG: Kyoto Encyclopedia of Genes and Genomes
mcl: Medium chain length
ML: Maximum likelihood
MW: Molecular weight
NCBI: National Center for Biotechnology Information
(NCBI) CDD: Conserved Domain Database
NMR: Nuclear magnetic resonance
PHA: Polyhydroxyalkanoates
PHB: Poly(3-hydroxybutyrate)
PHBV: Poly(3-hydroxybutyrate-co-3-hydroxyvalerate)
pI: Isoelectric point
PLA: Polylactic acid
PBS: Phosphate buffered saline
scl: Short chain length
TCA: Tricarboxylic acid
3-HV: 3-Hydroxyvalerate
3-HB: 3-Hydroxybutyrate
