# Supplementary material for: High natural PHA production from acetate in Cobetia sp. MC34 and Cobetia marina DSM 4741T and in silico analyses of the genus specific PhaC2 polymerase variant
Source: Microb Cell Fact. 2021 Dec 20;20:225. doi: 10.1186/s12934-021-01713-0 (PMC8686332; doi:10.1186/s12934-021-01713-0)
Supplement: Supplementary file 1 — Additional file 1: Figure S1. Tolerance- and requirement to sodium chloride in MMCY media supplemented with 2 % glucose at 30 °C, 200 rpm, shows highest specific growth rate in the range of 1-4 % for both strains (n = 6). Figure S2. Representative raw FTIR spectra for Cobetia sp. MC34 (blue) and Cobetia marina DSM4741T (orange) grown in MMCY and MMCY_2 media supplemented with 1.5 % glycerol, 4 % glucose, 4 % fructose or 20 g/l acetate + 8 mM sodium valerate (n = 1). Figure S3. Growth curves measured by optical density (OD600nm) for the strains Cobetia sp. MC34 (blue) and Cobetia marina DSM 4741T (orange) grown in MMCY media supplemented with 40 g/l glucose or fructose, respectively. The carbonyl-ester to amide band I-ester ratio obtained by FTIR is visualized as sphere area and indicate putative PHA production, which was determined at the end of the experiment by chloroform extraction and GC/MS quantification. Error bars represent standard deviation of OD600nm (n = 6). Table S1. List of Halomonadacea sequences used to construct the 16S- and ANI trees, and information about the strains. Table S2. Amino acid sequence similarities of the transporters, enzymes, transcriptional regulators and carbonosome proteins involved in PHA synthesis from acetate. Table S3. Fatty acid biosynthesis (FAS) and fatty acid degradation (ß-oxidation) genes found in Cobetia sp. MC34, derived from the genome annotation. [file 12934_2021_1713_MOESM1_ESM.docx]

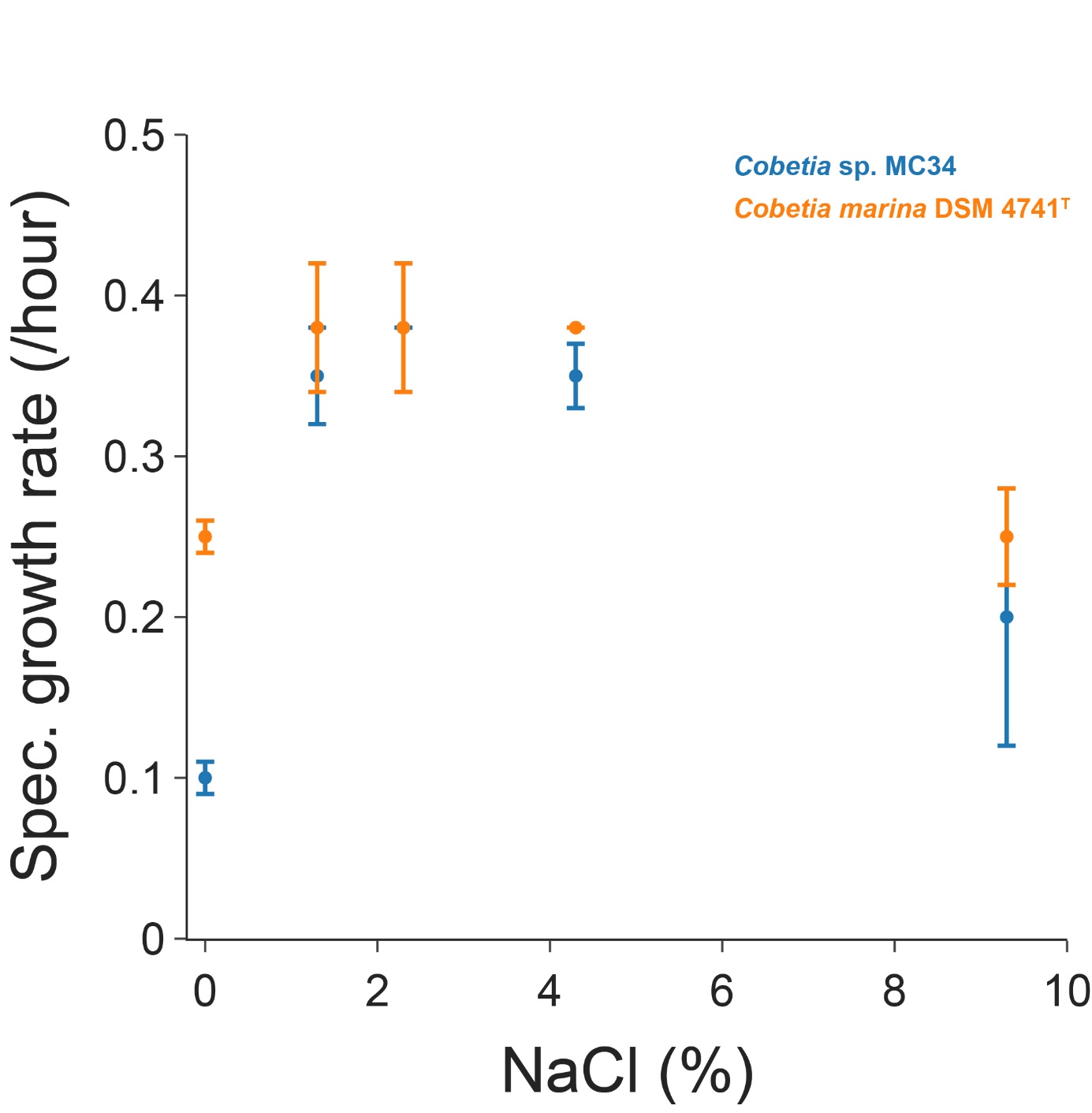


**Supplementary Figure 1.** Tolerance- and requirement to sodium chloride in MMCY media supplemented with 2 % glucose at 30 °C, 200 rpm, shows highest specific growth rate in the range of 1-4 % for both strains (n=6).

**
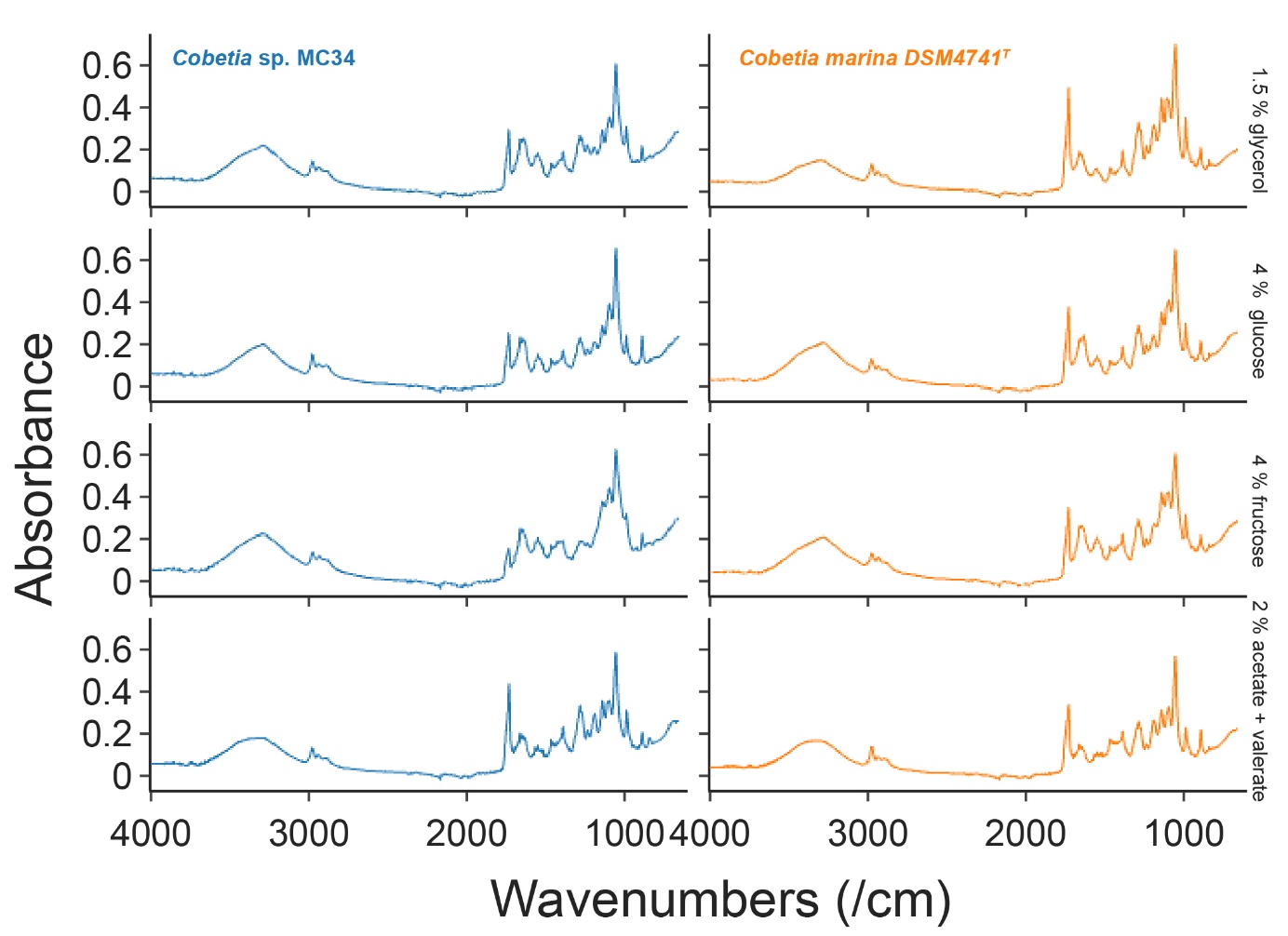
**

**Supplementary Figure 2**. Representative raw FTIR spectra for *Cobetia* sp. MC34 (blue) and *Cobetia marina* DSM4741^T^ (orange) grown in MMCY and MMCY_2 media supplemented with 1.5 % glycerol, 4 % glucose, 4 % fructose or 20 g/l acetate + 8 mM sodium valerate (n=1).


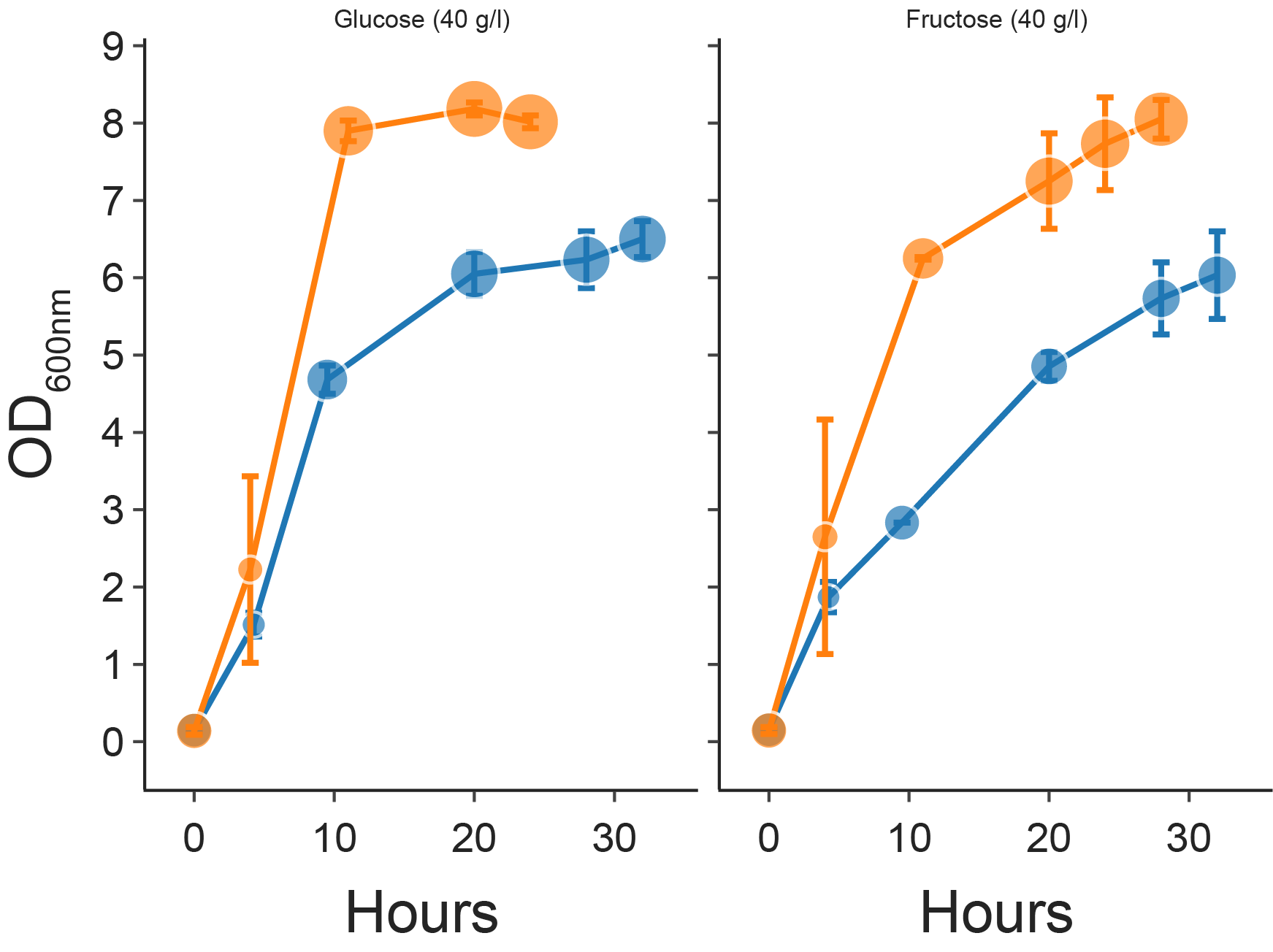


**Supplementary Figure 3**. Growth curves measured by optical density (OD_600nm_) for the strains *Cobetia* sp. MC34 (blue) and *Cobetia marina* DSM 4741^T^ (orange) grown in MMCY media supplemented with 40 g/l glucose or fructose, respectively. The carbonyl-ester to amide band I-ester ratio obtained by FTIR is visualized as sphere area and indicate putative PHA production, which was determined at the end of the experiment by chloroform extraction and GC/MS quantification. Error bars represent standard deviation of OD_600nm_ (n=6).

| **Supplementary Table 1.** List of *Halomonadacea* sequences used to construct the 16S- and ANI trees, and information about the strains. | | | | |
| --- | --- | --- | --- | --- |
| Strain | 16S acc. | Genome acc. (NCBI assembly) | Strain isolated from: | Reference |
| *Zymobactor palmae* | AF211871 (Silva) | - | Palm sap, Okinawa, Japan. | (1) |
| *Halomonas campisalis* LL4 MCM-B-365 | DQ077909.1 (Silva) | - | Alkaline lake, Lonar Lake, India. | (2) |
| *Halomonas salina* ATCC 49509 | NR_119189.1 | - | Hypersaline soil near the Mediterranean Sea coast near Alicante, Spain. | (3) |
| *Halomonas elongata* 1H9 ATCC 33173 | M93355.1 | - | Condensers at the  Antilles International Salt Go. facility on Bonaire,  Netherlands Antilles. | (4) |
| *Halomonas elongata* HEK1 | - | GCF_001678785.1 | Natural saltern of Runn of Kutch, Gujarat, India. | - |
| *Halomonas halophila* DSM 4770 | NR_116945.1 | - | hypersaline soils located near Alicante in southeast Spain. | (5) |
| *Chromohalobacter salexigens* | DQ789389 (silva) | - | Condensers at the  Antilles International Salt Go. facility on Bonaire,  Netherlands Antilles. | (6) |
| *Halomonas bluephagenesis* TD01 | JF340230.1 | GCA_000219565.1 | Salt lake,  Xinjiang, China. | (7) |
| *Halomonas campaniensis* LS21 | CP007757.1 | - | Sludge and plant debris samples, Dabancheng salt lake, Xinjiang, China. | (8) |
| *Halomonas boliviensis* LC1 | NR_029080.1 | - | Soil sample, lake Laguna Colorada, 4300 m above sea level, Bolivia. | (9) |
| *Halomonas profundus* | AJ876733 | - | Deep-sea vent field of the  Mid Atlantic ridge, depth 2291 m. | (10) |
| *Halomonas* sp. R5-57 | - | GCA_900070345.1 | Skin of the  red sea squirt Halocynthia papillosa collected from the  Barents Sea in Spring 2009. | (11) |
| *Cobetia* sp. AM6 | - | AP021868.1 | Exterior surface of the shell of an abalone sold in a fish market in Tokyo, Japan | Unpublished. |
| *Halomonas* sp. GDM18 | - | SJZC00000000.1 | Underwater soil sample, North Sea of Aberdeen, Scotland. | Unpublished. |
| *Cobetia* sp. L2A1 | - | GCA_009796845.1 | Brown algae, beach in the Arctic Ocean. | (12) |
| *Cobetia crustatorum* SM1923 | - | GCA_007786215.1 | Surface seawater,Kongsfjorden, Svalbard. | (13) |
| *Cobetia crustatorum* JO1 | - | GCA_000591415.1 | Jeotgal, a traditional Korean fermented seafood. | (14) |
| *Cobetia* sp. QF1 | **-** | GCA_002213105.1 | Crude oil-contaminated seawater of the Yellow Sea, China | (15) |
| *Cobetia* sp. UCD-24C |  | GCA_001306765.1 | Seagrass sediment,Garbage Beach, Woods Hole, Massachusetts, USA. | (16) |
| *Cobetia litoralis* | AB646234.1 | - | Sandy sediment sample collected at a depth of 1 m from the shore of the Sea of Japan, Russia. | (17) |
| *Cobetia amphilecti* KMM296 | - | GCA_000754225.1 | Mollusc Crenomytilusgraya-  nus, the Sea of Japan, Pacific Ocean. | (18) |
| *Cobetia* sp. MC34 | - | JADPPY000000000 | Swab from fork hole in storage through in maturation room for salted fish, Tromvik, Norway. | This study |
| *Halomonas* sp. SF2003 | - | GCA_003032495.1 | Brittany coastal sea water, France. | (19, 20) |
| *Cobetia* sp. MB87 | - | GCA_011319755.1 | Gut of sea cucumber, Hon Tre, Nha Trang Bay, Vietnam | (21) |
| *Cobetia* sp. ICG0124 | **-** | GCA_004006355.1 | Green algae “Ulva”, Aberystwyth, United Kingdom. | - |
| *Cobetia* sp. MM1IDA2H-1 | - | GCA_002916775.1 | Eulitoral intertidal pond at sea level, Valparaiso, Chile. | (22) |
| *Cobetia marina* DCM 4741^T^ | - | GCA_001720485.1 | Coastal sea sample near  Woods Hole (Massachusetts, USA). | (23) |
| *Cobetia pacifica* GPM2 | - | GCA_009931455.1 | Red algae *Pyropia tenera,* Namhae, (South) Korea | (17, 24) |
| *Cobetia sp. IU 180733JP01*  *(5-11-6-3)* | LC549335.1 | GCA_013374055.1 | Seaweed, Iwate, Ofunato Bay, Japan. | (25) |
| *Cobetia sp. 190790JP01*  *(5-25-*  *4-2)* | - | GCF_013374075.1 | Seaweed, Iwate, Ofunato Bay, Japan. | (25) |
| *Cobetia marina* T1 | **-** | GCA_005144735.1 | Hampton Beach, New Hampshire, USA. | - |
| *Cobetia marina* NBRC 15607^T^ | - | GCA_006540105.1 | Great Bay Estuary, Durham, New Hampshire, US. | (17) |
| *Cobetia* sp. MM1IDA2H-1 | - | GCA_002916775.1 | Eulitoral intertidal pond at sea level. Valparaiso, Chile. | (22) |
|  |  |  |  |  |

| **Supplementary Table 2.** Amino acid sequence similarities of the transporters, enzymes, transcriptional regulators and carbonosome proteins involved in PHA synthesis from acetate. | | | | |
| --- | --- | --- | --- | --- |
| **Gene** | ***Cobetia* sp. MC34 (locus tag)** | | ***Cobetia marina* DSM4741^T^ (%)** | ***Halomonas* sp. SF2003 (%)** |
| *phaA* | IZU87_07675 / (IZU87_02125) | 97.5 / 32.1 (95.7 )* | | 99.5 / 100 (42.4 )* |
| *phaB* | IZU87_12765 | 98.4 | | 99.6 |
| *phaC_1_* | **-** | 37.0 ** | | 37.9 ** |
| *pha*C_2_ | IZU87_04860 | 79.4 | | 93.7 |
| *phaP_1_* | IZU87_08295 | 86.7 | | 100 |
| *phaP_2_* | IZU87_04855 | 85.2 | | 99.3 |
| *phaR* | IZU87_03745 | 84.3 | | 100 |
| *scoA* | IZU87_09115 | 95.3 | | 100 |
| *scoB* | IZU87_09110 | 96.8 | | 99.6 |
| *bdhA* | IZU87_16460 | 94.6 | | 98.8 |
| *pta* | IZU87_07815 | 96.5 | | 99.7 |
| *bi-pta* | - | 81.5 *** | | 81.5 *** |
| *ackA* | - | 64.7 *** | | 64.7 *** |
| *acs* | IZU87_06405 | 95.8 | | 99.9 |
| *prpE* | IZU87_15385 | 92.7 | | 98.8 |
| *gltA* | IZU87_01890 | 98.1 | | 99.5 |
| *acnA / acnB* | IZU87_02425 / IZU87_05790 | 95.4 / 96.4 | | 99.7 / 99.9 |
| *AceA* | IZU87_14450 | 97.5 | | 99.1 |
| *AceB (glcB)* | IZU87_07285 | 93.9 | | 99.7 |
| Cation acetate symporter | IZU87_06430 | 99.5 | | 100 |

Percentage values are relative to *Cobetia* sp. MC34. * Relative to the ambiguous PhaA/fadA (IZU87_02125).
** For PhaC_1_ variants, the percentage values are relative to PhaC_2_ (IZU87_04860).
*** Percentage values are relative between *Cobetia marina* DSM4741^T^ and *Halomonas* sp. SF2003**.**

| **Supplementary Table 3**. Fatty acid biosynthesis (FAS) and fatty acid degradation (ß-oxidation) genes found in *Cobetia* sp. MC34, derived from the genome annotation. | | | |
| --- | --- | --- | --- |
| **Pathway** | **Gene name** | **Gene** | **Locus tag** |
| **FAS** | Acyl-carrier protein | *acpP* | IZU87_00125 |
|  | 3-hydroxyacyl-[acyl-carrier-protein] dehydratase (EC. 4.2.1.59, EC. 5.3.3.14) | *fabA* | IZU87_08605 |
|  | Beta-ketoacyl-ACP synthase I (EC. 2.3.1.41) | *fabB* | IZU87_11305, IZU87_08600, IZU87_10600, IZU87_10585 |
|  | ACP S-malonyltransferase (EC. 2.3.1.39) | *fabD* | IZU87_00135 |
|  | Beta-ketoacyl-ACP synthase II (EC. 2.3.1.179) | *fabF* | IZU87_00120 |
|  | 3-ketoacyl-(acyl-carrier-protein) reductase (EC. 1.1.1.100) | *fabG* | IZU87_00130, IZU87_10590, IZU87_10755 |
|  | Enoyl-[acyl-carrier protein] reductase I (EC. 1.3.1.9, EC. 1.3.1.10) | *fabI* | *IZU87_11845 |
|  | Trans-2-enoyl-CoA reductase family protein (EC. 1.3.1.9, EC. 1.3.1.44) | *fabV* | IZU87_14225 |
|  | 3-hydroxyacyl-ACP dehydratase (EC. 4.2.1.59) | *fabZ* | IZU87_02545 |
| **ß-oxidation** | Acetyl-CoA acyltransferase (EC. 2.3.1.16) | *fadA* | IZU87_11310 (IZU87_02125) |
|  | Enoyl-CoA hydratase, 3-hydroxyacyl-CoA dehydrogenase (EC 4.2.1.17, EC 1.1.1.35, EC 5.1.2.3, 5.3.3.8) | *fadB (fadJ)* | IZU87_11305, IZU87_05640, IZU87_05735, IZU87_05740, IZU87_06210, IZU87_05740 |
|  | ACP S-malonyltransferase (long-chain acyl-CoA synthetase) (EC 6.2.1.3) | *fadD* | IZU87_00135 |
|  | Acyl-CoA dehydrogenase (EC. 1.3.99.-) | *fadE* | IZU87_11485, IZU87_05730, IZU87_00670, IZU87_02145 |
|  | 2,4-dienoyl-CoA reductase (NADPH2). (EC.1.3.1.34) | *fadH* | IZU87_03580 |

***** Based on alignment with *fabI* annotated in *Halomonas* sp. SF2003.

**References**

1. Okamoto T, Taguchi, H., Nakamura, K., Ikenaga, H., Kuraishi, H., Yamazato, K. *Zymobacter palmae* gen. nov., sp. nov., a new ethanol-fermenting peritrichous bacterium isolated from palm sap. Arch Microbiol. 1993;160:333-7.

2. Joshi AA, Kanekar PP, Kelkar AS, Sarnaik SS, Shouche Y, Wani A. Moderately halophilic, alkalitolerant Halomonas campisalis MCM B-365 from Lonar Lake, India. J Basic Microbiol. 2007;47(3):213-21.

3. Chen Q, Zhang L, Li X, Liu S, Li D. Poly-β-hydroxybutyrate/ectoine co-production by ectoine-excreting strain Halomonas salina. Process Biochemistry. 2014;49(1):33-7.

4. Vreeland RH, Litchfield, C. D., Martin, S. E. L., Elliot, E. *Halomonas elongat,* a New Genus and Species of Extremely Salt-Tolerant Bacteria.pdf>. International Journal of Systematic Bacteriology. 1980;30(2):485-95.

5. Quesada E, Ventosa, A., Ruiz-Berraquero, F., Ramos-Cormenzana, A. *Deleya halophila* a new species of moderately halophilic bacteria. International Journal of Systematic Bacteriology. 1984;34(3):287-92.

6. Arahal DR, García, M. T., Vargas, C., Cánovas,, Nieto JJ, Ventosa, A. *Chromohalobacter salexigens* sp. nov., a moderately halophilic species that includes *Halomonas elongata* DSM 3043 and ATCC 33174. Int J Syst Evol Microbiol. 2001;51:1457-62.

7. Tan D, Xue YS, Aibaidula G, Chen GQ. Unsterile and continuous production of polyhydroxybutyrate by Halomonas TD01. Bioresour Technol. 2011;102(17):8130-6.

8. Haitao Yue, Chen Ling, Tao Yang, Xiangbin Chen, Yuling Chen, Haiteng Deng, et al. A seawater-based open and continuous process for polyhydroxyalkanoates production by recombinant Halomonas campaniensis LS21 grown in mixed substrates. Biotechnology for Biofuels. 2014;7(108s).

9. Quillaguaman J, Hatti-Kaul R, Mattiasson B, Alvarez MT, Delgado O. Halomonas boliviensis sp. nov., an alkalitolerant, moderate halophile isolated from soil around a Bolivian hypersaline lake. Int J Syst Evol Microbiol. 2004;54(Pt 3):721-5.

10. Simon-Colin C, Raguenes G, Cozien J, Guezennec JG. Halomonas profundus sp. nov., a new PHA-producing bacterium isolated from a deep-sea hydrothermal vent shrimp. J Appl Microbiol. 2008;104(5):1425-32.

11. Williamson A, De Santi C, Altermark B, Karlsen C, Hjerde E. Complete genome sequence of Halomonas sp. R5-57. Stand Genomic Sci. 2016;11(1):62.

12. Cha QQ, Wang XJ, Ren XB, Li D, Wang P, Li PY, et al. Comparison of Alginate Utilization Pathways in Culturable Bacteria Isolated From Arctic and Antarctic Marine Environments. Front Microbiol. 2021;12:609393.

13. Yu Y, Yang J, Zheng LY, Sheng Q, Li CY, Wang M, et al. Diversity of D-Amino Acid Utilizing Bacteria From Kongsfjorden, Arctic and the Metabolic Pathways for Seven D-Amino Acids. Front Microbiol. 2019;10:2983.

14. Kim MS, Roh SW, Bae JW. Cobetia crustatorum sp. nov., a novel slightly halophilic bacterium isolated from traditional fermented seafood in Korea. Int J Syst Evol Microbiol. 2010;60(Pt 3):620-6.

15. Guo P, Cao B, Qiu X, Lin J. Draft Genome Sequence of the Crude Oil-Degrading and Biosurfactant-Producing Strain Cobetia sp. QF-1. Genome Announc. 2018;6(3).

16. Alexiev A, Krusor ML, Jospin G, Lang JM, Eisen JA, Coil DA. Draft Genome Sequence of Cobetia sp. UCD-24C, Isolated from Roots and Leaves of the Seagrass Zostera marina. Genome Announc. 2016;4(2).

17. Romanenko LA, Tanaka N, Svetashev VI, Falsen E. Description of Cobetia amphilecti sp. nov., Cobetia litoralis sp. nov. and Cobetia pacifica sp. nov., classification of Halomonas halodurans as a later heterotypic synonym of Cobetia marina and emended descriptions of the genus Cobetia and Cobetia marina. Int J Syst Evol Microbiol. 2013;63(Pt 1):288-97.

18. Balabanova LA, Golotin VA, Kovalchuk SN, Babii AV, Shevchenko LS, Son OM, et al. The Genome of the marine bacterium Cobetia marina KMM 296 isolated from the mussel Crenomytilus grayanus (Dunker, 1853). Russian Journal of Marine Biology. 2016;42(1):106-9.

19. Thomas T, Elain A, Bazire A, Bruzaud S. Complete genome sequence of the halophilic PHA-producing bacterium Halomonas sp. SF2003: insights into its biotechnological potential. World J Microbiol Biotechnol. 2019;35(3):50.

20. Elain A, Le Fellic M, Corre YM, Le Grand A, Le Tilly V, Audic JL, et al. Rapid and qualitative fluorescence-based method for the assessment of PHA production in marine bacteria during batch culture. World J Microbiol Biotechnol. 2015;31(10):1555-63.

21. Cao TTH. Enzyme discovery for brown seaweed fucoidan modification. 2018.

22. Ibacache-Quiroga C, Ojeda J, Espinoza-Vergara G, Olivero P, Cuellar M, Dinamarca MA. The hydrocarbon-degrading marine bacterium Cobetia sp. strain MM1IDA2H-1 produces a biosurfactant that interferes with quorum sensing of fish pathogens by signal hijacking. Microb Biotechnol. 2013;6(4):394-405.

23. Cobet ABWJ, C.; Jones, G. E. The Effect of Nickel on a Marine Bacterium Arthrobacter marinus. Journal of General Microbiology. 1970;62:159-69.

24. Yusriyyah AA, Tasakka, A. C. M. AR., Latama, G. Identification of The Potential of Degrading Carrageenan in Red Algae *Kappapychus alvarezii* Symbiotic Bacteria. International Journal of Environment, Agriculture and Biotechnology. 2021;6(1).

25. Moriya H, Takita Y, Matsumoto A, Yamahata Y, Nishimukai M, Miyazaki M, et al. Cobetia sp. Bacteria, Which Are Capable of Utilizing Alginate or Waste Laminaria sp. for Poly(3-Hydroxybutyrate) Synthesis, Isolated From a Marine Environment. Front Bioeng Biotechnol. 2020;8:974.
